# Dermatologic manifestations of thyroid disease: a literature review

**DOI:** 10.3389/fendo.2023.1167890

**Published:** 2023-05-12

**Authors:** Benjamin Cohen, Adam Cadesky, Shuchie Jaggi

**Affiliations:** Department of Endocrinology, Donald and Barbara Zucker School of Medicine at Hofstra/Northwell, Great Neck, NY, United States

**Keywords:** thyroid hormone, skin, hypothyroidism, hyperthyroidism, thyroid cancer, dermatologic manifestations

## Abstract

**Introduction:**

Thyroid hormone is considered one of the key regulatory hormones for skin homeostasis. Multiple organs are affected by the release of peripheral thyroid hormones (T4 and T3) further regulating various functions at a cellular level. Specifically, skin is considered an important target organ in which the thyroid hormone has a significant impact. Multiple skin diseases are associated with thyroid hormone dysregulation. However, other striking dermatologic manifestations are seen in nails and hair as well. Hypothyroidism, hyperthyroidism, and thyroid cancer can have an array of cutaneous manifestations, and we present the recent updates in this field.

**Methods:**

A PubMed search was performed for updates in any new skin disease findings and treatments between 2010 and 2022. Research published in the past decade and previously known foundational skin findings associated with thyroid disease were presented in this review.

**Conclusion:**

Cutaneous manifestations of thyroid disease is one of the first notable signs of thyroid hormone dysregulation. This article reviews the recent updates on the thyroid and skin interplay, and it further discusses overt visible findings and various available treatment modalities.

## Introduction

Skin is the largest organ of the human body, and its primary function is protection from external trauma and infectious insults. It is a metabolically active organ and works primarily by self-renewing squamous epithelium by continuous differentiation. The superficial layer of the skin—the “epidermis”—replenishes every four weeks (1, 2). It is also the largest sensory organ of the body and can react to external stimuli such as heat, cold, touch, and pressure, which is essential for the body’s thermostat.

Being the largest organ of the body, the skin is an active immune organ and an important peripheral neuro-endocrine organ. It is closely related to endocrine systems and therefore contributes to the homeostasis of peripheral hormones (2).

Complex pathophysiology occurs with thyroid hormone action on the skin, especially in the case of ongoing overt thyroid hormone dysregulation. Gross cutaneous manifestations are usually the first sign of thyroid hormone imbalance in which it plays an integral part in sustaining natural function. Here we present a review of thyroid hormone pathology and its interplay with the skin, manifesting in form of various cutaneous pathologies.

## Pathophysiology

The skin is composed of two main components – the dermis and epidermis – and they are separated by the basement membrane. The epidermis has four layers; the bottom layer is stratum basale, and directly above it is the spinous and granular layers with the stratum corneum as the outermost layer. The epidermis receives nutrients from blood vessels in the underlying dermis. Hair follicles and sebaceous glands are embedded in the dermis (2). The stratum basale contains basal keratinocytes that are responsible for the continuous replenishment of the epidermis, though only 15 percent are actively involved in this process (3). Proliferation is limited to the basal layers, and during differentiation, basal keratinocytes move outwards to the surface of the skin. While undergoing this process, they produce stratum corneum, responsible for the barrier function.

The pilo-sebaceous glands contain hair follicles which are embedded in the dermis. At the end of the hair follicle is a hair bulb, which is made from specialized mesenchymal cells. Epidermal stem cells are produced in a specific region of the hair follicle called the bulge. The epidermal stem cells oversee the continuous turnover of the epidermis. For skin homeostasis maintenance, a balance between proliferation and differentiation is necessary (4).

The dominant circulating thyroid hormone, T4, is the precursor hormone. It is later converted t T3 by two intracellular thyroid hormone deiodinases (D1 and D2). The role of the third deiodinase (D3) is to convert T4 to inactive reverse T3. The inactivating D3 is not expressed in most peripheral tissues, but the activity (primarily expressed in the epidermis) has been seen *in vivo* human skin. D2 activity has been demonstrated in cultured human fibroblasts (5).

Keratins are the most important proteins produced by keratinocytes which are seen in multiple layers of the epidermis. The cytoskeleton of the basal keratinocytes is formed by the filaments of keratin 5 and 14 (6). The binding of T3 to thyroid receptors regulates the gene expression in the keratinocytes, confirming that thyroid hormone plays an important role in keratinocyte proliferation. *In-vitro* analysis has suggested that T3 depleted keratinocytes have lower levels of the plasminogen activator, an enzyme seen in the corneocyte shedding process (7). Human skin fibroblast cultures showed that other genes such as RAS oncogene family (RAB3B), Collagen (COLVIA3-CVIIIA1), hypoxia inducible factor (HIF)-1A, a calcineurin inhibitors ZAKI4a and AKR family that are responsive to thyroid hormone were found to play a role as well in skin as well (8).

Thyroid hormone action on the skin is primarily mediated through the thyroid hormone receptor (TR). All three widely recognized thyroid hormone binding isoforms have been identified in skin tissues. Thyroid hormone signaling occurs from interaction with TR and T3-TR complexes increasing or inhibiting the expression of target genes by binding specific TH response elements (TREs) within chromatin. The two isoforms of TH receptors, *thra* and *thrb*, are respectively encoded in the THRA and THRB. They act both as a positive and negative regulator in the skin (9–11). Thyroid hormone receptors have been found in skin fibroblasts, sebaceous glands, smooth muscle cells, and Schwann cells, and many other thyroid hormone responsive genes have been found in the skin. T3 has been shown to stimulate growth of both epidermal keratinocytes and dermal fibroblasts. The thyroid hormone also increases cell proliferation of fibroblasts, which later inhibits hyaluronate synthesis (12). During embryogenesis, the thyroid hormone has a vital role in establishing the barrier function of the epidermis by increasing activity of the cholesterol sulfate cycle (6).

The thyroid hormone accelerates barrier formation, and in hypothyroidism, this can hinder the epidermal barrier function (4). Hyaluronic acid is the major glycosaminoglycan that accumulates in myxedema which can involve multiple organs (13). Extravascular accumulation along with insufficient lymphatic drainage occurs due to transcapillary escape of albumin (14, 15). The appearance of skin is cool and pale because of the dermal mucopolysaccharides and water content being affected in hypothyroidism.

The hair cycle is normally comprised of anagen, catagen, and telogen. Anagen is the growth phase of the hair cycle. Roughly 90% of scalp hairs are anagen at any given time, and the cycle lasts for each hair anywhere between two and six years. Catagen is the transformation phase of the hair cycle when hair production stops and the lower portions of the hair follicle regresses. Catagen typically lasts less than three weeks and accounts for less than 1% of scalp hair at any given point in time. The final phase of the hair cycle is telogen—the resting phase characterized by club hairs that are ready for shedding. Around 10% of scalp hairs are estimated to be in telogen at any given time. After the hair falls out, the hair cycle recommences with anagen (16). Alopecia, a common finding in hypothyroidism is caused bychanges in hair cycle due to a lack of thyroid hormone, and increased hairs in telogen phase. Additionally, diffuse or partial alopecia can be seen along with loss of the lateral third of the eyebrows. In the hypothyroid state, nails can become fragile, brittle, thickened, and slow growing.

In hyperthyroidism, the most frequent thyroid hormone–related epidermal disease is hyperhidrosis. which occurs in patients with Graves’ disease. Thyrotoxic skin is generally warm, smooth, and moist compared to euthyroid subjects. Hair in thyrotoxicosis is often fine and soft. A diffuse nonscarring alopecia may also be observed. Common nail changes include development of a concave contour with distal onycholysis (17).

Dermatologic changes seen in hyperthyroidism and hypothyroidism in skin, hair, and nails will be described in more detail in the upcoming sections (See **Table 1**). Other skin manifestations seen in thyroid cancer will also be further elucidated.

**Table 1:** A table of dermatologic conditions associated with hypothyroidism and hyperthyroidism.

	Hypothyroidism	Hyperthyroidism
**Skin changes**	•Coarse, thin, scaly skin (Xerosis) •Pallor •Carotenemia •Edema	•Warm, smooth, moist skin
**Associated skin conditions**	•Palmoplantar Keratoderma •Acquired ichthyosis •Myxedema •Vitiligo	•Pre-Tibial Myxedema •Thyroid Acropachy
**Hair changes**	•Dry, brittle, coarse hair	•Fine Hair
**Associated hair conditions**	•Trichodystrophy •Alopecia •Telogen Effluvium •Madarosis	•Alopecia •Pili Annulati
**Nail changes**	•Coarse, dull, thin, brittle nails	•Shiny, soft, friable nails
**Associated nail conditions**	•Terry’s Nails	•Plummer’s nails •Lindsay’s nails •Onycholysis

## Hyperthyroidism and skin disease

Hyperthyroidism has several causes (including, most commonly, Graves’ disease), followed by other causes such as thyroiditis, toxic multinodular goiter, toxic adenoma, and iatrogenic thyroid hormone use (18). Thyroid hormone is tightly regulated in the blood stream *via* the hypothalamic-pituitary-thyroid (HPT) axis (1). However, when a pathologic process such as hyperthyroidism occurs, the biochemically active thyroid hormone will be higher than normal, leading to abnormalities that can occur in nearly all parts of the body. Skin, being the largest organ in the human body, is a prime target for thyroid hormone action; it is involved in fetal epidermal differentiation, barrier formation, hair growth, wound healing, keratinocyte proliferation, and keratin gene expression (5).

The classic associations of skin pathology with hyperthyroidism include thin but not atrophic skin, scalp hair with a downy texture and scalp thinning (19), hyperhidrosis, facial flushing, and palmar erythema (20). This section will focus on thyroid dermopathy (pretibial myxedema) and thyroid acropachy, and it will touch on some of the other associated skin diseases with thyrotoxicosis including eczema (21), Plummer’s nails (onycholysis in the setting of hyperthyroidism) (22, 23), generalized pruritus, eczematous dermatitis, and thinning of the scalp (18).

In recent years, hyperthyroidism has been found to manifest in common, unusual, subtle, or simply bizarre cutaneous manifestations that challenge our traditional understanding. Knowing different presentations of hyperthyroidism related skin disease can help point a clinician to the correct diagnosis. Treatment varies based on whether the skin manifestation is a direct result of thyroid hormone action or its extra-thyroidal manifestations.

## Skin

### Pretibial myxedema

Pretibial myxedema or thyroid dermopathy is a well-known manifestation of Graves’ disease, commonly seen with Graves’ ophthalmopathy. Occurring in a 4:1 female to male ratio, it usually presents alongside hyperthyroid features. Tobacco is thought to be a risk factor in developing both thyroid dermopathy and thyroid acropachy (24). It is believed to occur by TSH stimulation in skin fibroblasts (25). While most commonly confined to the pretibial area, hence the name “pretibial myxedema,” (26) it can occur in other areas of the body, including a patient’s shoulders, upper back, and dorsal surface of their hands or feet (24, 27). Given the significant immune process required for its development, pretibial myxedema usually occurs after the onset of other hyperthyroid manifestations, such as ophthalmopathy (28).

Pretibial myxedema can be visualized as well-demarcated pink or purple atrophic or transparent papules (24). It can resemble a ‘peau d’orange’ appearance when depression of the hair follicles presents as non-pitting edema which can manifest in a nodular or tubular form (25). Lymphedema and elephantiasis from severe lymphatic obstruction is the extreme manifestation of pretibial myxedema. A recent case from 2018 demonstrates this manifestation in a 49-year-old male (found to be biochemically euthyroid with very elevated TSH receptor antibodies) with severe elephantiasis and bilateral exophthalmos after having previously received radioactive iodine therapy for thyrotoxicosis (29). Multiple other recent cases of patients with Graves’ disease who developed thyroid dermopathy after radioactive therapy—ranging from pretibial myxedema (30, 31) to elephantiasis (32) —have been reported in the literature.

**Figure 1:**
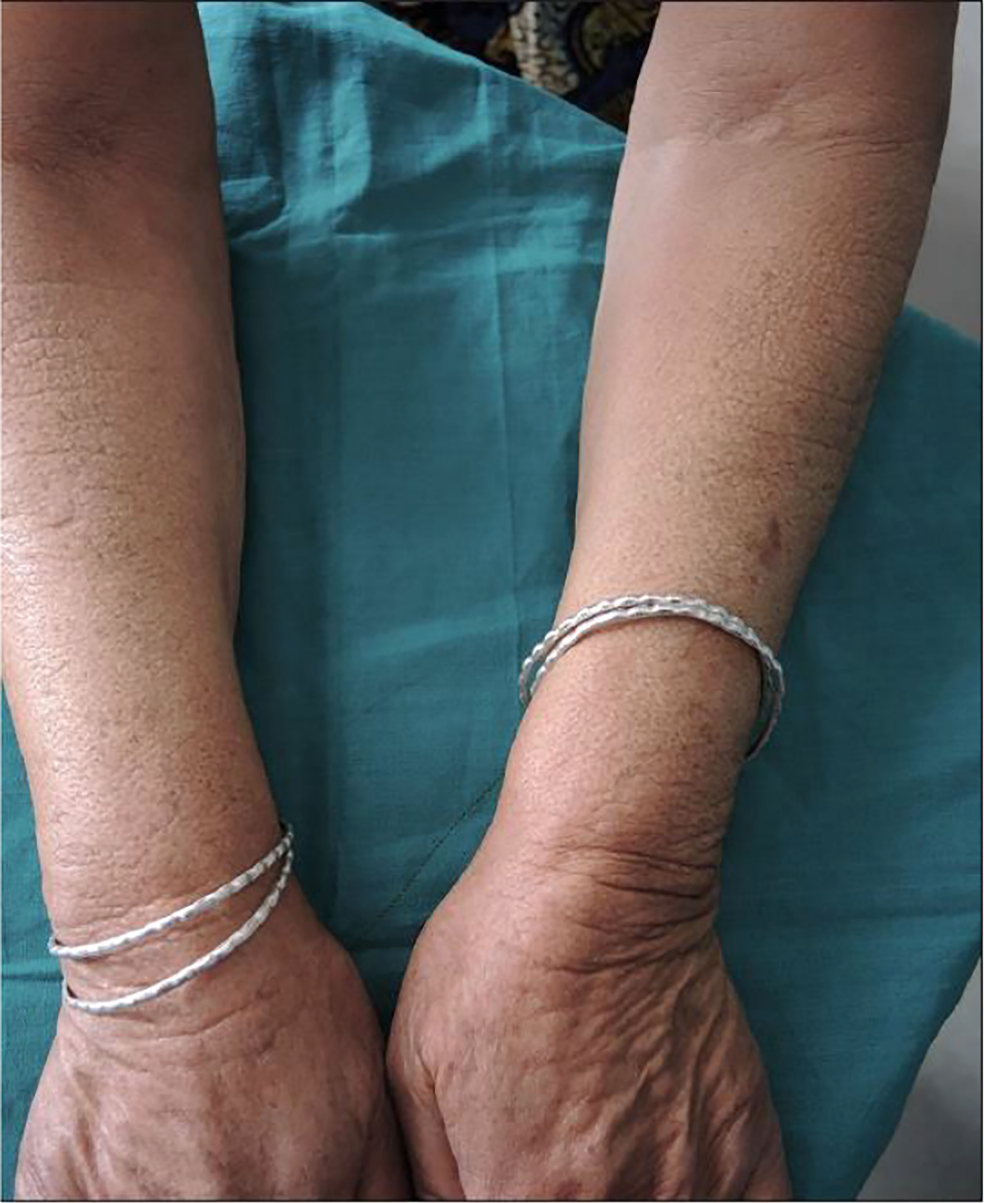
Indurated plaques having peau de’orange appearance on extensors of forearms.

While TSH receptor antibodies are thought to be involved in thyroid dermopathy, a case of thyroxine-induced pre-radial myxedema in 2019 was described in a 70-year-old woman who presented with yellow plaques on the extensor surface of her bilateral forearms with the aforementioned ‘peau d’orange’ appearance (as seen in **Figure 1**). She previously had a distant history of a goiter treated with partial thyroidectomy with resultant hypothyroidism requiring levothyroxine therapy. Since the patient was biochemically hyperthyroid, the patient was thought to have had levothyroxine induced pre-radial myxedema, both an unusual cause and location for thyroid dermopathy (33 **)**.

The patient’s clinical picture is often sufficient to diagnose thyroid dermopathy. Skin biopsy can be used as a confirmatory test in more nuanced cases. Skin biopsy will demonstrate mucin and glycosaminoglycan deposition that spread collagen bundles in the dermis and stain Alcian blue (25). The skin biopsy is helpful in differentiating between similar presentations of lichen myxedematosus (characterized by grouped soft papules on the face and arms with circumscribed mucin deposits) (34, 35) and scleromyxedema (diffuse erythema and skin thickening with papules with proliferation of fibroblasts) (36). The deposited mucin promotes retention of fluid, which causes lymphatic compression leading to lymphedema (37). Of note, if a patient has negative TSH receptor antibodies, alternative diagnoses should be strongly considered (25) however, cases have been described, one receiving thyroxine therapy for history of subtotal thyroidectomy for multinodular goiter (38) and others without any history of thyroid disease or treatment (39, 40).

Treatment—such as antithyroid medications, radioactive iodine ablation, and surgery are aimed at addressing the underlying thyroid disease. However, treatment with systemic or topical corticosteroids have also been used with mixed benefits (25). While myxedema is visually concerning, it is generally not painful, and is often treated due to cosmetic concerns. Some cases may be self-resolve. In theory, having a total thyroidectomy or thyroid ablation could help improve thyroid dermopathy *via* elimination of the antigen source in Graves’ disease (41).

Some theorize that the addition of corticosteroid therapy may be beneficial in patients with Graves’ ophthalmopathy (42) and that it may also be applicable to dermopathy. Given obesity associated lymphedematous mucinosis (43), another possible approach to assist in dermopathy management is weight loss (44). Compressive therapy with bandages or wraps to help move lymphatic circulation has also been used (26). Octreotide therapy has been attempted in a case of severe thyroid dermopathy without success (43). Given the diffuse mucinosis, intralesional hyaluronidase has also been theorized and used with appropriate effect at decreasing pretibial myxedema given as 150 U/ml—1ml injected in 10 equal aliquots at week 0, 6, 12, and 18 (45).

The more advanced the dermopathy, the less likely a specific therapy will work. One mode of treatment is topical therapies which include triamcinolone acetanilide with an occlusive dressing in conjunction with nightly clobetasol propionate (46), as well as 0.2% fluocinolone acetonide (47 **)** therapy covered with plastic wrap (47, 48). Intralesional corticosteroid injections have been shown to be beneficial in nodular pretibial edema and can be used in combination with topical corticosteroids to achieve remission (49). Topical therapy will need to be applied nightly for a prolonged period of four to ten weeks with decreasing frequency once improvement has begun. A case of cutaneous myxedema in a Graves’ disease patient in 2014 was successfully treated with a daily dose of 15 mg of carbimazole and 40 mg/ml of intralesional triamcinolone every three weeks (50). More recently, a 2018 case of pretibial myxedema presenting as elephantiasis was managed successfully using debulking surgery, skin grafts, and intralesional injections of 10 mg triamcinolone followed by further surgery (29). However, surgical management has not been well studied in the literature. Another recent case of a patient with severe elephantiasis related to thyroid dermopathy responded to treatment with rituximab and plasmapheresis with rapid improvement in symptoms (51). Lastly, teprotumumab has been used in refractory pretibial myxedema patients related to Graves’ disease with ophthalmopathy dosed at 10 mg/kg intravenously followed by 20 mg/kg intravenously every three weeks for seven additional infusions with improvement in thyroid dermopathy (52, 53).

Thyroid dermopathy has proven to be a manifestation of thyroid disease that can point a physician to hyperthyroidism. Its many presentations continue to intrigue clinicians and presentations such as thyroxine induced myxedema challenge our traditional understanding. As more cases become apparent, our knowledge will only continue to grow resulting in better treatments for thyroid dermopathy.

### Thyroid acropachy

While related to thyroid dermopathy, thyroid acropachy is a distinct entity and a very uncommon manifestation of autoimmune thyroid disease. It consists of a combination of digital swelling, nail clubbing in association with thyroid ophthalmopathy and dermopathy (24). Its incidence is less than 1% and can occur in biochemically euthyroid or hypothyroid patients whose thyrotoxicosis had previously been treated (54, 55). It most often presents with thyroid dermopathy and ophthalmopathy but rarely can be seen isolated without dermopathy. It is thought to be the last manifestation following ophthalmopathy and dermopathy and may occur in around 20% of these patients (24). Acropachy represents one of the most severe manifestations of thyroid disease.

With findings including nail-clubbing and digital-swelling, the differential for thyroid acropachy is wide and includes pulmonary osteoarthropathy and other systemic conditions, highlighted in **Table 2** (56). A series of differences can help distinguish thyroid acropachy from alternative diagnoses. Patients with thyroid acropachy have positive TSH receptor antibodies differentiating it from other diseases. Thyroid acropachy nearly always involves ophthalmopathy and dermopathy, which may occur in alternative diagnosis (55). Pain is not common with thyroid acropachy whereas it can occur in other diseases (19). In terms of location, thyroid acropachy rarely affects long bones compared to other systemic disorders with similar findings (56). On X-ray imaging, thyroid acropachy shows an asymmetric sub-periosteal reaction that may be spiculated or frosty in appearance compared to pulmonary osteoarthropathy, which usually has a symmetric laminar periosteal profile (57). Ultimately, skin biopsy can help confirm the diagnosis showing findings consistent with pretibial myxedema that are similar to those with thyroid acropachy (24).

**Table 2:** Differences between thyroid acropachy and other disorders associated with clubbing and pulmonary osteoarthropathy.

	Thyroid acropachy	Other systemic disorders
Associated signs	Ophthalmopathy, and dermopathy	Depends upon system involved
Pain	Rarely	Frequently
Radiological sign	Involvement of long bones rare	Common
Periosteal reaction	Symmetrical	Asymmetrical
	Characteristic subperiosteal spiculated, frothy, or lacy appearance	Laminal periosteal proliferation
Pathogenesis	TSH Receptor Antibodies in high titer	Absent
Pathology	Autoimmune, trapping of platelets, increase glycosaminoglycan and fibroblast proliferation	Tapping of platelets
Spontaneous remission	Up to 50%	Infrequent
Treatment	No specific treatment	Specific treatment available depends upon pathology

TSH, Thyroid Stimulating Hormone.

In 2019, a case of thyroid acropachy occurred in a biochemically euthyroid Graves’ disease patient. A 52-year-old male with a history of Graves’ disease who had a total thyroidectomy and bilateral orbital decompression on levothyroxine presented with marked myxedematous changes. The patient was biochemically euthyroid but had bilateral soft tissue swelling, digital clubbing, and irregular periosteal bone formation of his digits with lower extremity swelling consistent with thyroid acropachy (58).

Interestingly, up to 50% of patients can achieve spontaneous remission of the dermopathy with no specific guidelines for treatment (24, 25). Often focusing on the concomitant ophthalmopathy and dermopathy, systemic immunosuppressive therapy and local corticosteroid therapy (25 **)** have been used as treatments. Octreotide injections and localized radiation therapy have been attempted for non-abating pulmonary osteoarthropathy but are of unclear benefit in patients with thyroid acropachy (44, 59). A case of a 45-year-old male with Graves’ thyrotoxicosis found to have skin biopsy proven thyroid acropachy was described in 2012. The patient was ultimately managed with carbimazole and propranolol to treat the hyperthyroidism, though the dermopathy lesions were not treated as they did not bother the patient (59).While a rarer entity than thyroid dermopathy, acropachy is an equally important manifestation to consider when diagnosing patients with hyperthyroidism as it indicates a late-stage presentation.

### Other skin findings in hyperthyroidism

Jaundice, a condition with a wide differential, can be a presenting manifestation of hyperthyroidism either as a direct cause of thyrotoxicosis, manifestation of medical treatment of hyperthyroidism, or a presentation of an associated autoimmune condition. The link between the thyroid gland and the liver is that the thyroid hormone is essential for liver function and, conversely, the liver is important for its metabolism (60). The exact mechanism of thyrotoxicosis induced liver injury is not fully known but may be related to ischemic injury or direct effects on the hepatocytes (61). A proposed mechanism involves increased demand for hepatic oxygenation secondary to hyperthyroidism without a proportionate increase in hepatic blood flow that could cause a reduction of bile transport leading to cholestasis (62). Separately, autoimmune hepatitis, which can cause jaundice has been linked to Graves’ disease (63).

In a 2013 paper of three case reports, jaundice was described as the presenting skin finding of Graves’ disease. Each of them described a case of a young female who presented with jaundice and signs of hyperthyroidism being diagnosed with Graves’ disease who were all successfully treated with carbimazole and propranolol leading to full resolution of symptoms (64). Since 2013, multiple other cases of patients with Graves’ disease presenting with jaundice have been illustrated with resolution of jaundice from treatment of thyrotoxicosis with thionamides as well as cholestyramine, radioactive iodine therapy, glucocorticoids, potassium iodide, and radioactive iodine therapy (65–68).

These cases demonstrate that jaundice can be an initial symptom of thyrotoxicosis and should alert a clinician to check thyroid function tests in the appropriate clinical context. Making it even more challenging is that anti-thyroid medications such as methimazole and propylthiouracil, which can treat hyperthyroidism-related jaundice can also cause transaminitis leading to jaundice. Knowing when to hold and when to treat the jaundice with anti-thyroid medications or use alternative therapies such as cholestyramine or glucocorticoids will continue to be a challenge in the future.

Another disease that has may be associated with Graves’ disease is Henoch-Schonlein Purpura (HSP) (69). This was demonstrated by the case of a 31-year-old female who presented with a hand tremor, weight loss, pruritis, and a palpable purpuric rash. The patient was found to be biochemically hyperthyroid and was treated with methimazole, only to develop worsening of the purpuric lesions. Skin biopsy was obtained, which was suggestive of Henoch-Schonlein Purpura. Colchicine 10 mg daily and prednisolone 100 mg daily were started with improvement of lesions but tapering of steroids could not resolve it. Treatment with 10 mCi of radioiodine was used with disappearance of purpuric lesions. The patient was eventually placed on levothyroxine after development of hypothyroidism with recurrence of skin lesions after overtreatment and resultant hyperthyroidism. Decreasing the dose led the patient to be biochemically euthyroid with resolution of cutaneous lesions. This suggested multiple possible associations of Graves’ disease to HSP either as a side effect of methimazole, thyrotoxicosis itself, or iatrogenic thyrotoxicosis from levothyroxine treatment (69).

Given that HSP has been a known side effect of anti-thyroid medications such as propylthiouracil (70), definitively identifying thyrotoxicosis as a direct cause has been difficult. This association may continue to push physicians to using alternative therapies such as radioactive iodine therapy to best manage these patients.

## Nails

### Onycholysis

Onycholysis is an interesting cutaneous manifestation, which happens *via* distal separation of the nail plate from the nail bed (71) that can occur through hyperthyroidism (called Plummer’s nails) (22, 23) or other mechanisms including psoriasis (72), certain cancer drugs (73), fungal and bacterial infections (74), and direct trauma (71). Plummer was the first to describe Plummer’s nails when he drew attention to this manifestation of onycholysis in patients with hyperthyroidism (75). Its treatment therefore focuses on the underlying cause.

There have been several cases of onycholysis as a presenting symptom of hyperthyroidism. A case of onycholysis that preceded the diagnosis of Graves’ disease by seven years was described in 2019. It was initially thought to be a fungal nail infection but eventually Graves’ disease was diagnosed with positive TSH receptor antibodies and biochemical hyperthyroidism. Treatment with thiamazole 10 mg three times a day, inosine 0.2 g three times a day, leucogenum 20 mg three times a day, potassium chloride 0.5 g twice daily, and recombinant human granulocyte colony-stimulating factor 50 μg intravenously every three days (for neutropenia) was initiated with significant improvement in his symptoms (76).

**Figure 2:**
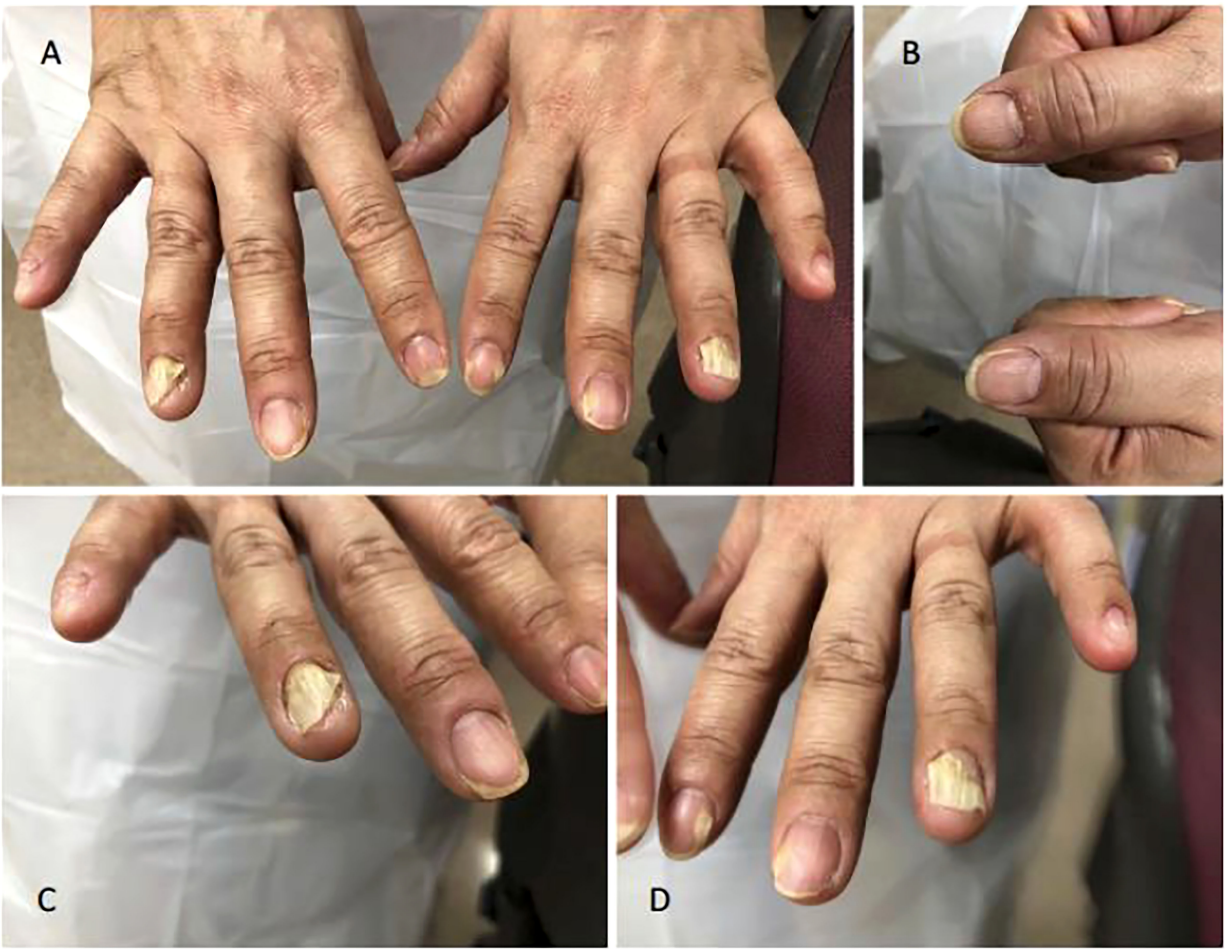
Plummer’s Nails demonstrated on **(A)** Bilateral hands, **(B)** Thumbs, **(C)** Right Hand, and **(D)** Left Hand.

Conversely, onycholysis can present in Graves’ disease patients who are biochemically hypothyroid as described in 2018. A 46-year-old women who presented with signs and symptoms of hypothyroidism such as bradycardia and delayed reflexes found to be biochemically hypothyroid with positive TSH receptor antibodies demonstrated characteristic Plummers nails as seen in **Figure 2** (22). This would be a unique presentation for someone who was clinically hypothyroid with Graves’ disease presenting with onycholysis.

Given the multitude of causes of onycholysis, it is important for the physician to use clinical judgment when ascertaining the etiology and obtaining thyroid function tests where thyroid disease is suspected.

### Half-and-half nails

**Figure 3:**
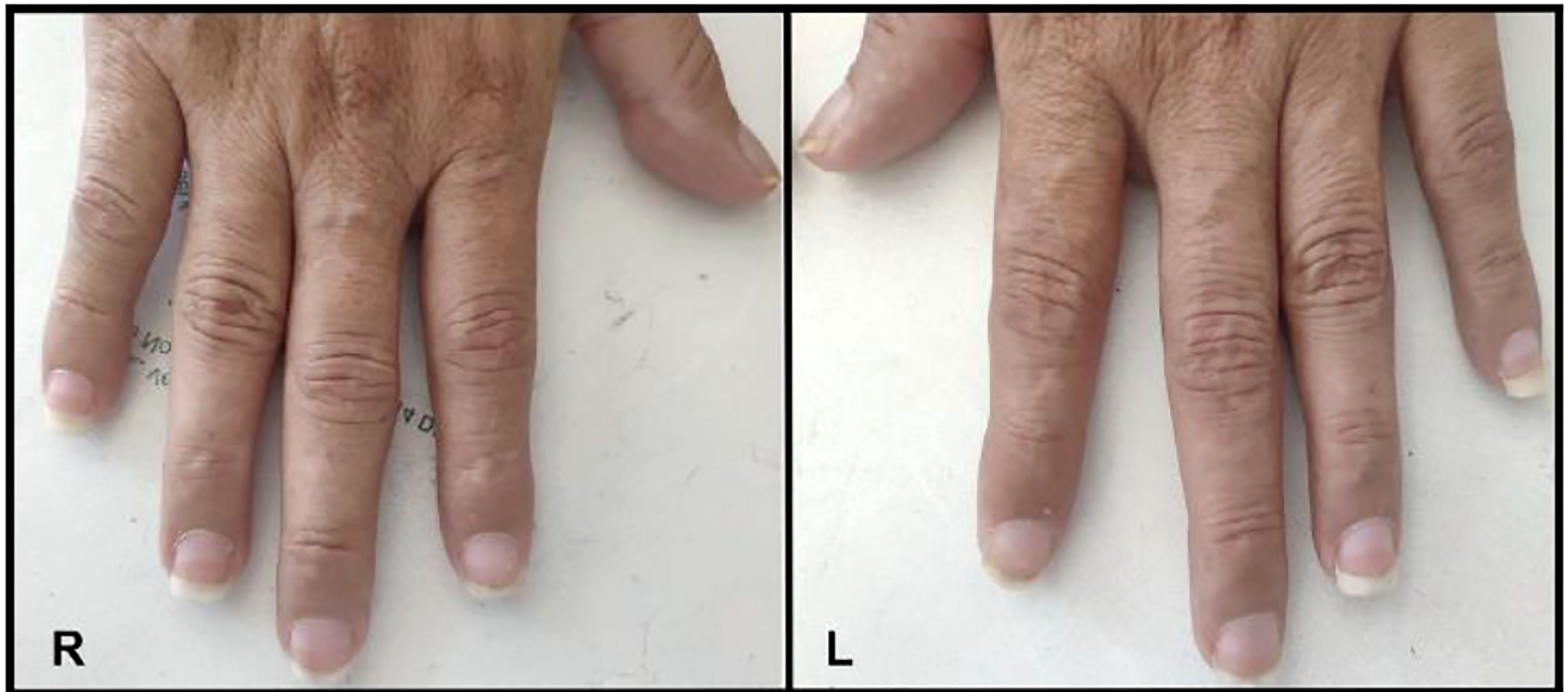
Half-and-half nails shown on the right and left hand.

An unusual phenomenon called half-and-half nails or Lindsay’s nails where the proximal nail is white, with at least 20% of the distal nail being more pinkish-brown, has been recently described in a patient with Graves’ disease (77). The term was first coined by Philip Lindsay in 1967 when he described these nail changes in patients with azotemia whom it usually presents in (78). The mechanism is not yet known however, to help identify it with histology, melanin deposits can be seen on the ungual plate (79). It was recently seen in a 48-year-old woman who presented with altered mental status found to be in thyroid storm with acute kidney injury with a reddish-brown distal nail plate discoloration with proximal white banding as seen in **Figure 3** (77). She was initiated on methimazole and lugol solution with resolution of nail color after one month of treatment. The association of half-and-half nails is usually with patients who have chronic renal insufficiency not acute kidney injury, thus this new and recent case described shows the novelty of Lindsay nails found in a patient with acute kidney injury and Graves’ disease.

## Hair

### Alopecia areata

Alopecia areata (AA) is an autoimmune condition that results in hair loss without scarring that is well known to be associated with autoimmune diseases such as Graves’ disease or Hashimoto’s thyroiditis and is thought to have a multifactorial autoimmune process (80). The etiology of AA is unclear and is thought to be similar to the T cell mediated response in Graves’ or Hashimoto’s disease but instead, is believed to be an antibody response against hair follicle proteins (81, 82). The lesions can be disfiguring and can cause psychological stress with low self-esteem and morbidity making it more frustrating (83). Skin biopsy of these lesions would reveal perifollicular inflammation with lymphocyte infiltration surrounding anagen follicles (84). A unique case in 2013 described a female patient previously diagnosed with Hashimoto's thyroiditis on levothyroxine who developed hyperthyroidism and alopecia areata. Graves’ disease was subsequently diagnosed with positive TSH receptor antibodies. She was treated with propylthiouracil and topical corticosteroids, which resolved her AA symptoms (85).

In general, AA can be difficult to manage with a general lack of effective therapies used to treat the condition. A challenging case to treat described in 2021 was that of a 35-year-old female who presented with alopecia universalis (rare variant of AA where all body hair is lost) and hyperthyroidism features with positive thyroid stimulating immunoglobulin and anti- thyroperoxidase (TPO) antibodies. This patient was started on daily prednisolone 40 mg, topical fluocinolone acetonide, minoxidil 2% and carbimazole with minimal response. Ultimately hydroxychloroquine and azathioprine were added as adjunct therapy with discontinuation of oral steroids after one week. One month later, the patient developed improvement in hair growth in the scalp region (86). Similar cases where hydroxychloroquine and azathioprine as treatment for AA have also been described elsewhere in the literature (87, 88). Other proposed second line agents that have been described in the literature include minoxidil, phototherapy, cyclosporine, sulfasalazine, methotrexate, and new biologics (89, 90).

### Pili annulati

Another disease of the hair that has been more recently linked with hyperthyroidism is called pili annulati (PA) or ringed hair, an autosomal dominant disorder characterized by its appearance and first described in the mid 1800s (91). It is a disease in which an alternating pattern of light and dark bands or rings give the hair a speckled or banded appearance. Under light microscopy, the pattern is reversed with bands being seen as shaded air-containing spaces, created by the reflection of light on the keratin lined surface of the hair (92, 93). Under electron microscopy, it can be seen as air-filled cavities that were initially filled with fluid distinguishing it from normal hair fibers (90).

**Figure 4:**
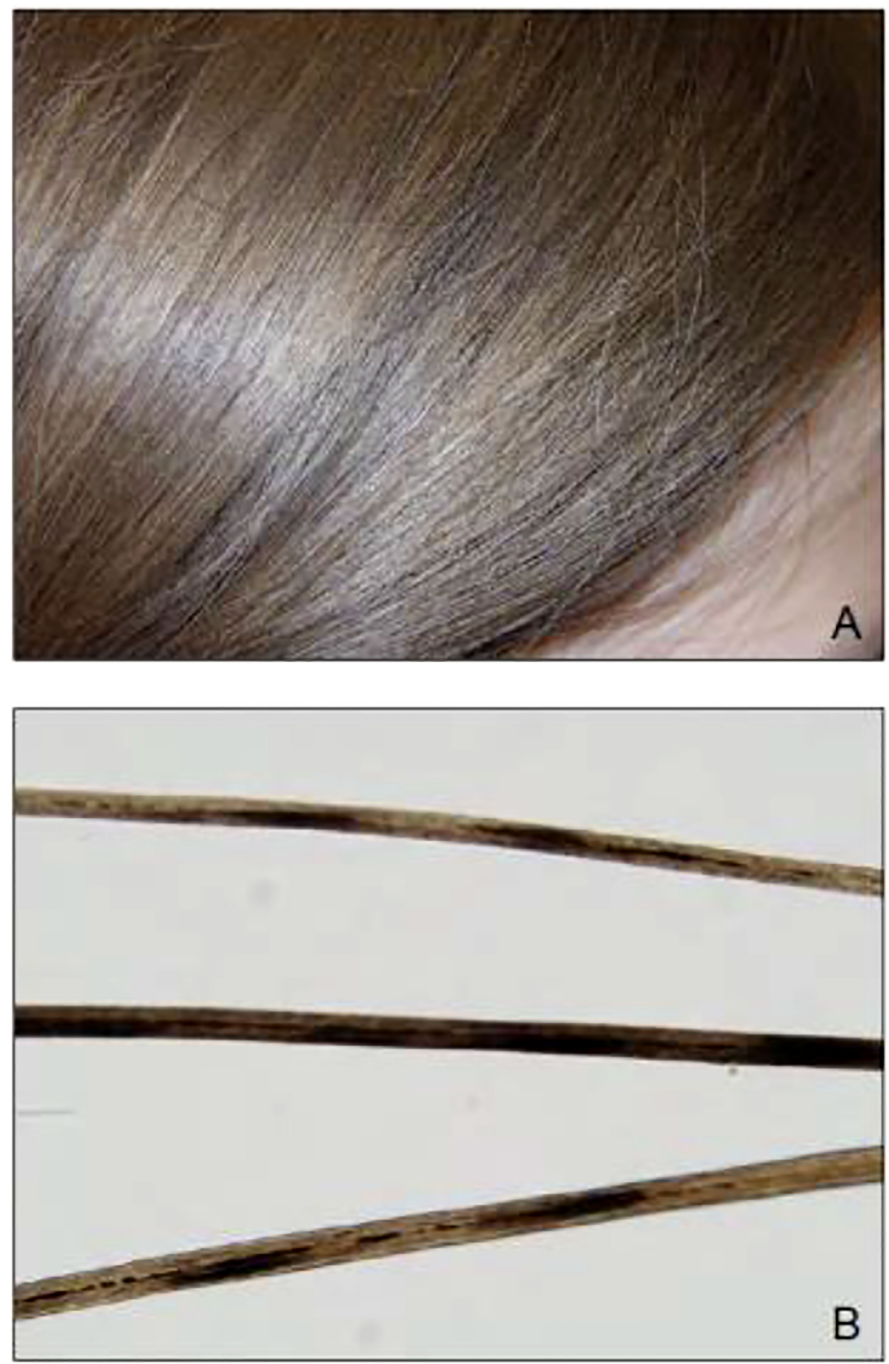
**(A)** Blonde-brown scalp hair with a speckled appearance on physical examination of a 10-year-old girl. **(B)** Light microscopy revealed a banded appearance on her scalp hair with dark air-containing spaces in the cortex.

Pili annulati was observed in a case in 2013 where a 10-year-old female with Graves’ disease who developed post-ablative hypothyroidism on levothyroxine presented with mild diffuse hair loss (94). Her scalp hair had a blonde-brown speckled pattern with banding seen on light microscopy as shown in **Figure 4** (95). Her hair loss eventually resolved within a month without intervention, though the PA remained.

While pili annulati and Graves’ disease co-occurring together is rare, an even more infrequent presentation is that of a patient with Graves’ disease who developed both AA and PA as described in 2012 (95). In this case, a 17-year-old female with a history of IgA deficiency and AA presented to a physician after failing treatment of AA with topical high-potency steroids for three months. On physical exam she demonstrated characteristic findings of both AA and PA of the uninvolved hair. Thyroid function tests were obtained with a suppressed TSH, and positive TSH receptor antibody confirming a diagnosis of Graves’ disease. Tiamazol 5 mg daily was initiated, systemic therapy for AA was deferred given her known immunodeficiency, and topical tacrolimus was applied with little effect, resulting in the progression to alopecia totalis (complete hair loss of the scalp) (95).

While AA and PA have been linked to Graves’ disease, the mechanism is still unknown, paving the way for further investigation into this fascinating association. Treatment for these conditions varies widely in the literature and will continue to evolve as more knowledge is gained from treating these conditions.

## Other skin diseases associated with hyperthyroidism

### Chronic idiopathic urticaria

The concomitant presence of chronic urticaria (CU) and autoimmunity was first described in 1907 (96) but has been clinically linked to autoimmune thyroid disease since at least 1983, when anti-microsomal antibodies were shown to be present in a portion of patients suffering from chronic idiopathic urticaria (CIU) (97). Urticaria is defined as chronic if it occurs daily for more than 6 weeks. Hashimoto’s disease has been diagnosed in up to 30% of patients with chronic urticaria in the literature (98, 99). Much is still hypothesized and theorized in terms of the connection between chronic urticaria and autoimmune thyroid disease, though a lot is still unknown (100).

Chronic urticaria is defined as the occurrence of erythematous, pruritic wheals of differing sizes, with or without edema of the deeper skin layer (angioedema) (101, 102). The development of pruritic wheals is complex involving cutaneous mast cell activation regulating vasoactivity. It involves a lymphocyte and granulocyte mediated sensitivity reaction evolving as urticaria *via* proinflammatory mediators such as cytokines and chemokines (103). Circulating IgG antibodies with high affinity for IgE receptor and less commonly IgE or IgG antibodies linking to 34 kD alpha subunit of the IgE receptor have been reported as a cause, with autoantibodies leading to activation of mast cells and basophiles (104–106).

Hashimoto’s disease is thought to be linked to chronic urticaria due to the presence of anti-thyroid antibodies and autoimmunity being associated with more severe CU (106). Cross reactivity of thyroid autoantibodies and other autoantibodies has been found to possibly lead to CU, though lack of evidence has been seen *in vivo* on mast cell and basophil activation (107, 108). Hyperthyroidism is thought to lead to chronic urticaria through activation of kinins, which are substances formed in response to body tissue injury leading to vasodilation and smooth muscle contraction (98). Other proposed mechanisms include the inflammatory response in the thyroid gland leading to a more generalized inflammatory state and lowering the threshold of mast cell stimulation (108), as well as TSH mediated production of proinflammatory cytokines (109). Chronic urticaria is uncommonly a patient’s sole presenting complaint when uncovering Graves’ disease but was seen in a recent case in 2020 (110).

Thyroid hormone replacement therapy has been reported as a treatment for thyroid autoantibody positive patients who have CU with some efficacy (111), leading to subsequent recommendation as a therapeutic modality (112). In 2017, a 39-year-old woman with Hashimoto’s thyroiditis and chronic urticaria was treated with levothyroxine 25 µg daily and cetirizine 10 mg daily with resolution of urticarial symptoms after 4 weeks (96). Antithyroid drugs have also been suggested in the literature to help achieve remission in patients with chronic spontaneous urticaria (113). In addition, steroids have been used with some success, however patients can require additional treatments such as radioactive iodine therapy to help achieve remission (114). A 2014 case of a 27-year-old male who was not able to achieve remission of urticaria with dexamethasone for several months until Graves’ disease was discovered. The patient was able to achieve remission and discontinuation of dexamethasone after 31.1 mci of I-131 radioactive iodine therapy was used (114).

**Figure 5:**
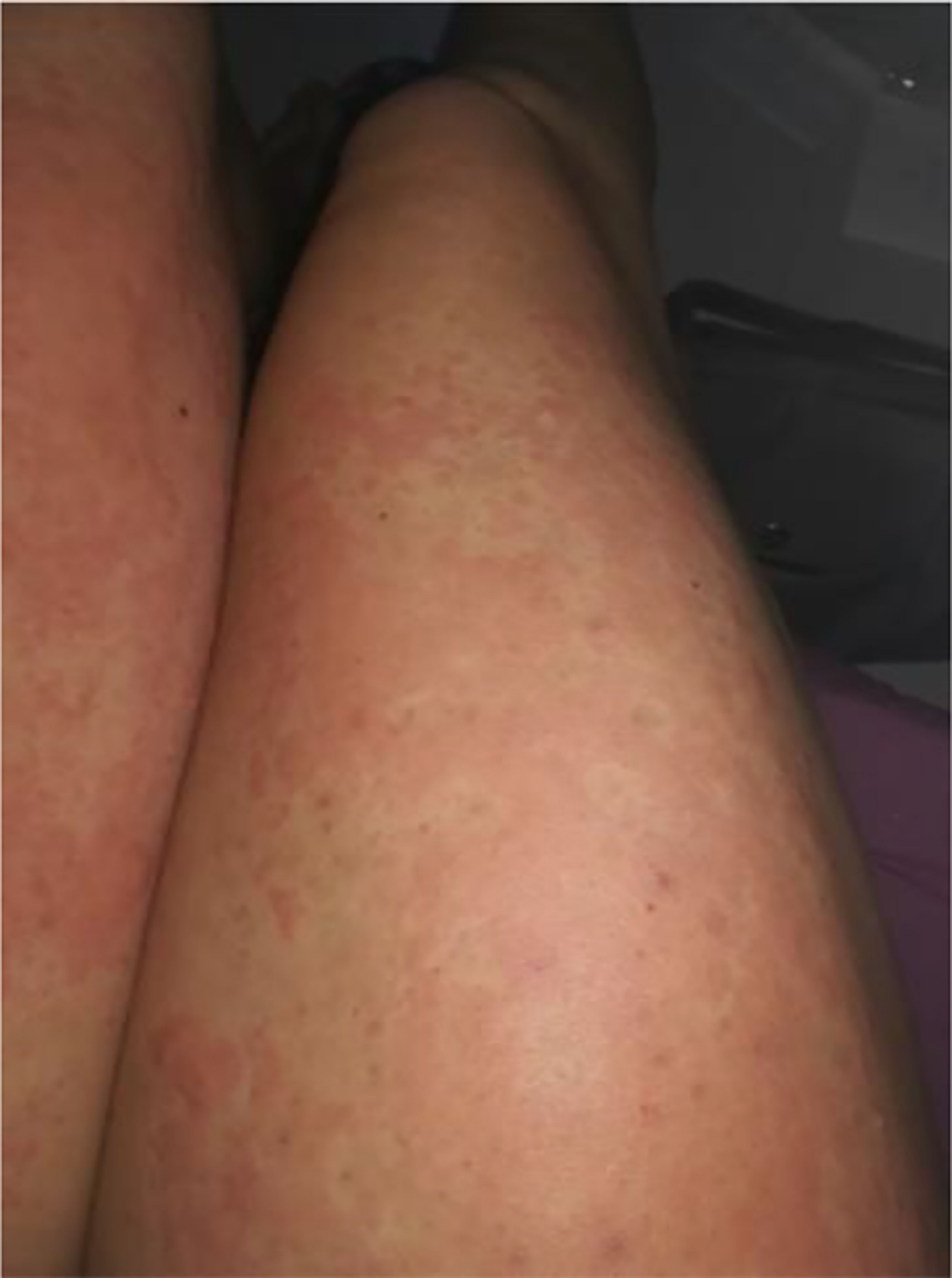
Lower and upper extremity chronic idiopathic urticaria.

While radioactive iodine therapy has been shown to be effective, total thyroidectomy has been used as another tool to manage chronic spontaneous urticaria in patients with Graves’ disease. This was demonstrated in the recent case in 2022 of a 35-year-old women with chronic idiopathic urticaria and facial angioedema as seen in **Figure 5** who was found to be biochemically euthyroid with positive anti-TPO and anti-thyroglobulin (TG) antibodies. She had previously been given multiple medications for treatment including oral corticosteroids and anti-histamines as well as levothyroxine, with little effect. She underwent total thyroidectomy with complete resolution of urticaria and angioedema three days post operatively (115).

### Acute urticaria

Another entity linked to autoimmune thyroid disease is acute urticaria, defined as urticaria for less than 6 weeks duration (116), unlike chronic idiopathic urticaria which lasts longer than 6 weeks. In 2020, a 23-year-old female who had just been diagnosed with Graves’ disease being treated with carbimazole presented with a spreading urticarial rash. She was found to have significantly elevated TPO antibodies and normal TSH receptor antibodies. Carbimazole was stopped as a possible side effect leading to urticaria, and the patient was treated with high dose chlorphenamine and topical menthol cream with hydrocortisone. The patient’s symptoms eventually resolved. It was unclear whether the patient’s hyperthyroidism or carbimazole led to the rash, but both may have played a role (117). This demonstrates the challenges in managing Graves’ disease patients when the effective treatments also have side effects that may resemble the skin manifestations themselves. Additional therapeutic modalities that have been used to treat CU because of the frequent presence of autoantibodies include plasmapheresis and intravenous immunoglobulin with appropriate response (118, 119).

### Urticarial vasculitis

A more threatening urticarial disease related to Graves’ disease is urticarial vasculitis. Urticarial vasculitis normally presents with purpuric urticarial lesions that would demonstrate a cutaneous leukocytoclastic vasculitis on histopathology in contrast to acute or chronic urticaria which would show edema in histopathology (101, 102). Furthermore, the lesions from urticarial vasculitis characteristically last longer than 24 hours. The lesions can be differentiated from urticaria from other causes such as infection or related to food as they tend to be associated with a burning sensation and longer duration (120). Furthermore, thionamide-induced antineutrophil cytoplasmic antibody-associated vasculitis such as with propylthiouracil is another important entity to recognize that can present similarly but would require discontinuation of thionamide therapy (121). A recent case in 2022 described a 29-year-old woman who presented with a painful and pruritic rash with signs and symptoms of hyperthyroidism including biochemical evidence of hyperthyroidism with positive TPO antibodies, positive TSH receptor antibodies and low complement C3 levels. Skin biopsy from an abdominal lesion confirmed leukocytoclastic vasculitis. She was initiated on methylprednisolone 40 mg daily, propylthiouracil 200 mg daily, and propranolol 30 mg daily with no recurrence of urticarial vasculitis after three months of treatment (122).

Urticaria has been shown to be linked to thyroid disease in many forms including chronic urticaria, acute urticaria and urticarial vasculitis. The mechanisms linking each one continues to evolve as our knowledge in the field grows. Increasing our identification of these diseases coexisting together will help improve our understanding and result in the development of novel therapies to help combat both thyroid disease and urticaria.

## Hypothyroidism and skin disease

Hypothyroidism is a common endocrinopathy encountered by a variety of subspecialists and primary care providers outside the field of endocrinology. Hypothyroidism occurs when the thyroid is unable to make sufficient thyroid hormone. The most common cause of hypothyroidism worldwide is iodine deficiency. In the United States, iodine is supplemented in various dietary items (majority in table salt), and therefore iodine deficiency is a highly uncommon cause for hypothyroidism. The most common cause of hypothyroidism in the United States is the autoimmune condition Hashimoto’s thyroiditis. Other causes include thyroidectomy, radiation therapy, fibrosis from infiltrative diseases, pituitary dysfunction, and thyroiditis. Certain drugs can also lead to hypothyroidism most notable are thionamides, lithium, and amiodarone. With the advent of new immunotherapy as a potential treatment for various malignancies, the PD1 inhibitors and CTLA4 inhibitors have also been identified as causes for either primary or secondary hypothyroidism (123).

According to the National Health and Nutrition Examination Survey (NHANES 3) that was performed between 1988 and 1994, hypothyroidism was estimated to effect 4.6% of the US population. Of that 4.6%, 0.3% presented with overt hypothyroidism and 4.3% with subclinical hypothyroidism. Cutaneous manifestations have been reported in both forms of hypothyroidism_1_. In addition, hypothyroidism is more prevalent in women than in men. Hypothyroidism is diagnosed with simple blood tests assessing the TSH, free thyroxine (FT4) and triiodothyronine (T3). TSH is produced by the anterior pituitary and stimulates the thyroid to make thyroid hormone. The majority of thyroid hormone produced is thyroxine (T4), and in lesser quantities triiodothyronine (T3), which is the active thyroid hormone that plays a vital role in regulating our metabolism and activity of every organ system. While the majority of thyroid hormone that is produced is T4, the body is able to convert to T3 in the peripheral circulation. Symptoms of hypothyroidism develop due to low levels of circulating T3. In primary hypothyroidism, the TSH is elevated and the FT4 is low. Many symptoms of hypothyroidism may be subtle, but more severe forms of hypothyroidism, namely myxedema coma, can present as life threatening. A variety of dermatologic manifestations of hypothyroidism affect the skin, hair, and nails. These findings vary from patient to patient and often depend on the severity and duration of the underlying hypothyroidism (124).

Thyroid hormone acts directly on skin *via* the thyroid hormone receptor, which has been detected in epidermal keratinocytes, skin fibroblasts, hair arrector pili muscle cells, other smooth muscle cells, sebaceous gland cells, vascular endothelial cells, Schwann cells, and a number of other cell types that make up the hair follicle. Thus, thyroid hormone interacts and helps regulate all layers of the skin including the epidermis, dermis, hair, nails, and sweat glands (1).

## Skin

### Xerosis cutis

The most common epidermal change in hypothyroidism is xerosis cutis, or excessively dry and scaly skin, most notably on the extensor surfaces of our extremities and including the palms and soles. It is the most common dermatologic manifestation in patients with hypothyroidism. In one study of 460 patients, xerosis cutis was identified in 57.16% of patients, followed by diffuse hair loss (46.09%), altered skin texture (31.74%), coarse scalp hair (29.35%), and facial edema (28.69%) (125).

The cause for xerosis cutis in patients with hypothyroidism currently remains unclear despite multiple hypotheses. First, under histologic examination of the skin in hypothyroidism, the epidermis appears thinned and hyperkeratotic. *In vitro* conditions demonstrated T3 as a key regulator of epidermal growth and keratin gene expression. When T3 is bound to the TR, keratin gene expression is deactivated, thus in hypothyroid states where T3 levels are low, hyperkeratosis can result, leading to the appearance of scaly skin (126). Another hypothesis is that xerosis develops due to decreased production of essential sterols. Thyroid hormone can accelerate skin barrier formation by increasing the activity of the enzyme cholesterol sulfate, which in turn, induces expression of the skin protein filaggrin in epidermal keratinocytes. Filaggrin then leads to accumulation of collapsed corneocytes, the final differentiated form of the keratinocyte, leading to the creation of the skin barrier and the stratum corneum (127). In addition, hypothyroidism disrupts formation of the Odland bodies, which help to form the epidermal permeability barrier (12). Finally, the third hypothesis for xerosis in hypothyroidism has been attributed to hypohidrosis due to decreased eccrine gland secretion (2). Additionally, some evidence notes that euthyroid patients that use topical T3 as treatment for xerosis and hyperkeratosis may be beneficial and thus provides supportive evidence of the role of T3 in the development of xerosis (128).

Xerosis, while common most often can be treated with topical agents including alpha-hydroxyl acids, ointments with unctuous materials such as petroleum or lanolin, liquid emulsions, oils, and urea (129).

### Palmoplantar keratoderma

**Figure 6:**
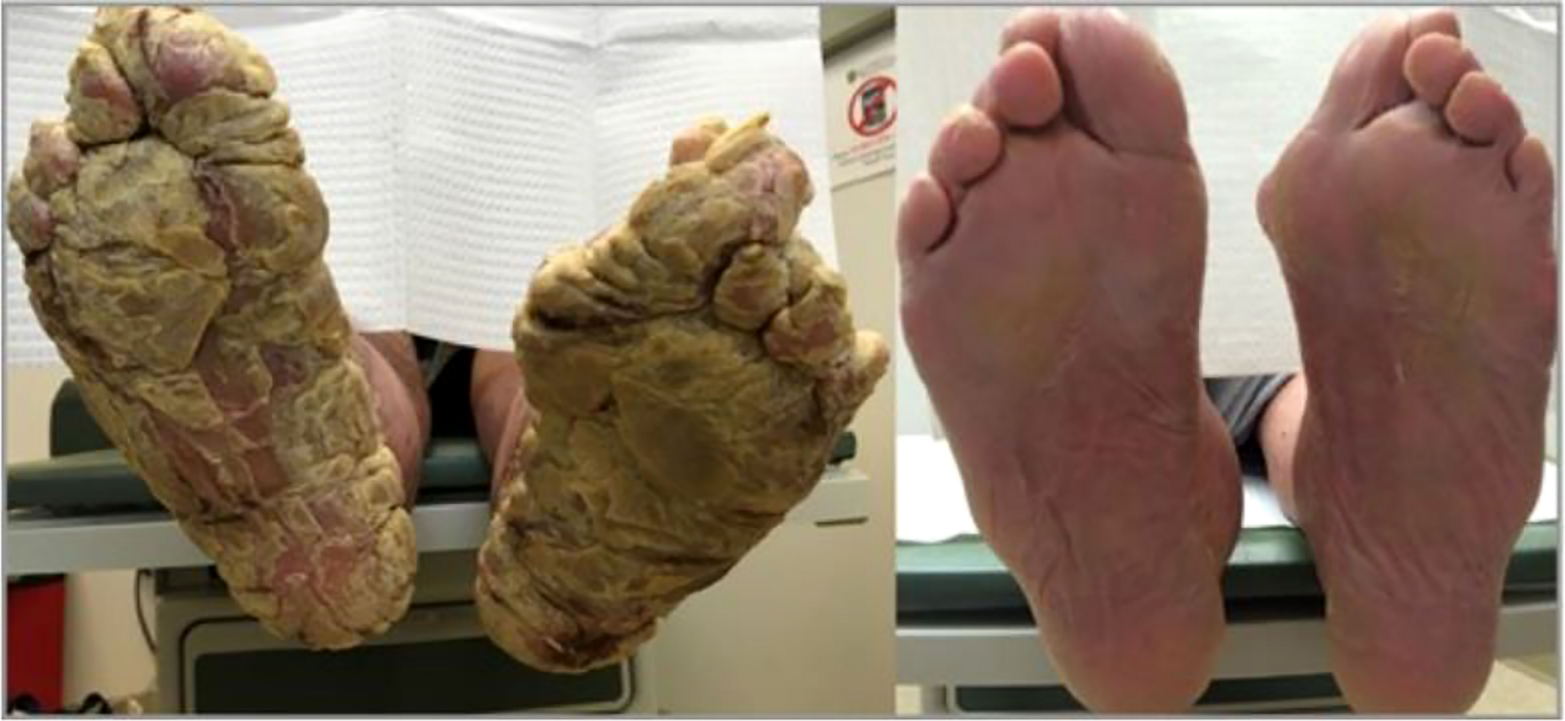
Above is an image of acquired palmoplantar keratoderma. The image on the left is upon diagnosis. The image on the right is the same patient 4 months after normalization of her thyroid function tests.

Patients with xerosis may also present with more severe skin disease and the typical topical agents used as treatment may not be as effective. One example of this is acquired palmoplantar keratoderma (**Figure 6**) (130), a condition with thickened skin on the palms and soles. There have only been a few case reports of acquired palmoplantar keratoderma. These case reports showed a predominance of middle-age to elderly patients with worsening keratoderma on the soles compared to the palms (131). Keratoderma appeared as a verrucous thickening of the skin. Typical treatment of palmoplantar keratoderma involves the use of keratolytics, topical or systemic retinoids, and in some cases corticosteroids (132). In each of the reported cases of acquired palmoplantar keratoderma due to hypothyroidism, these standard treatments were not effective. Despite this, treatment of hypothyroidism did result in resolution of the condition as demonstrated in a case report of a 67-year-old female with severe palmoplantar keratoderma who was found to have a TSH of 238 mIU/mL who had complete resolution of her condition after 4 months of treatment with levothyroxine (130). Overall, the etiology of why this condition develops is unclear but may be related to dysregulation of lipids in the stratum corneum (131).

### Acquired ichthyosis

**Figure 7:**
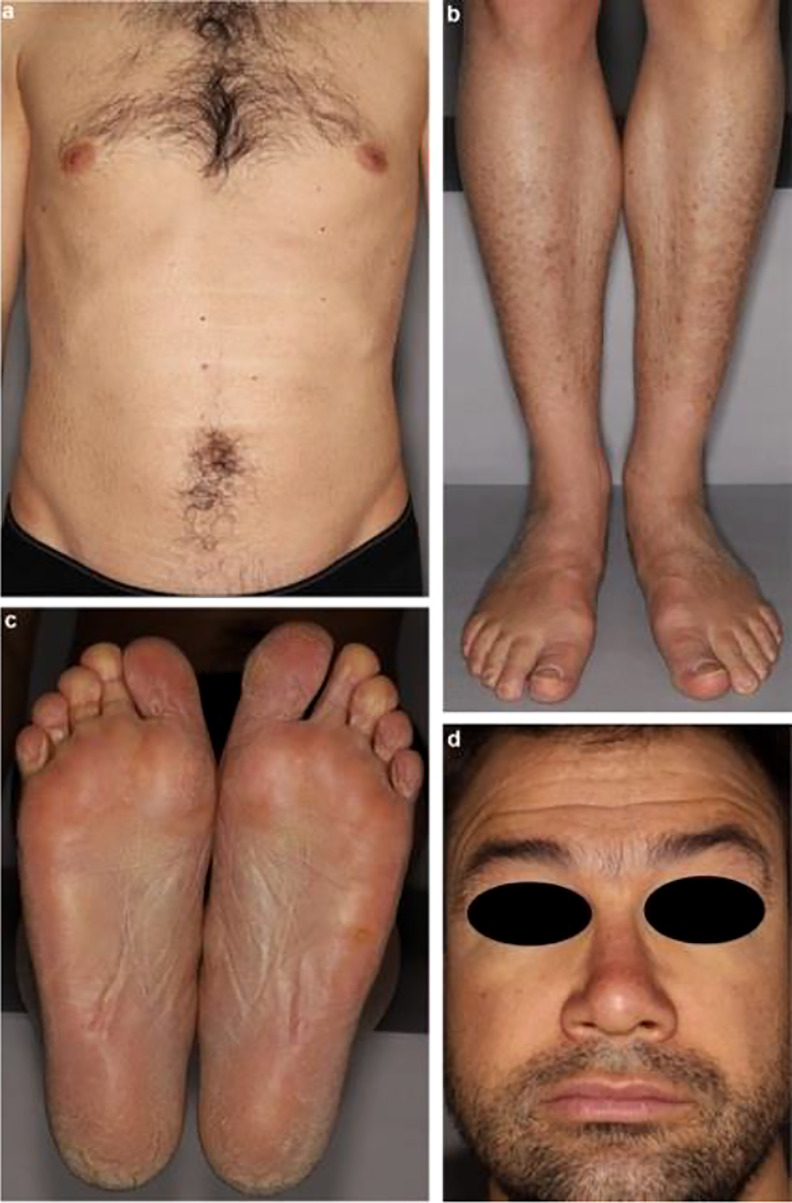
Ichthyosis clinical presentation: **(A)** and **(B)** diffuse acquired ichthyosis with large scales adhering to the skin; **(C)** orange palmoplantar keratoderma; **(D)** swollen face.

Another example of patients with xerosis presenting with more severe skin disease is when patients develop acquired ichthyosis. In acquired ichthyosis patients develop large fish-like thick scales that are adherent to the skin (**Figure 7**) (133). One case report of a 45-year-old man who presented with signs and symptoms of hypothyroidism and development of acquired ichthyosis, palmoplantar keratoderma, and myxedema. TSH was elevated to 132 mIU/mL and FT4 and total T3 were undetectable. The patient’s skin condition resolved with treatment of hypothyroidism (133). Like palmoplantar keratoderma, the specific cause for acquired ichthyosis in hypothyroidism is unclear but may be due to dysfunction of epidermal lipid metabolism. It may be a finding more common in severe and prolonged hypothyroidism (124).

## Pigmentary changes

### Pallor

Aside from the dry and scaly appearance, the skin in hypothyroidism may also vary in color and pigmentation. The most common manifestation of pigmentary change is pallor. This develops due to cutaneous vasoconstriction, which develops as the body’s metabolic rate drops in order to try and preserve core body temperature. Increased deposition of mucopolysaccharides and water also alters the refraction of light (12). In addition, hypothyroidism may cause anemia, which can also contribute to the development of pallor (134). The most severe form of pallor in hypothyroidism may mimic livedo reticularis (124).

### Carotenemia

Skin in hypothyroidism may develop a yellowish hue due to carotenemia. This yellowish hue may also be mistaken for jaundice as seen in hyperbilirubinemia. One way to differentiate these two causes for yellow discoloration of the skin is that carotenemia will spare the sclera, whereas jaundice will not. Carotenemia develops in hypothyroidism due to deficient conversion of carotene into vitamin A. Thyroid hormone is an antagonist of vitamin A and is also helps mediate the rate of catabolism and conversion from carotene to vitamin A. Therefore, in patients with hypothyroidism and insufficient thyroid hormone the process of conversion of carotene to vitamin A is slowed and may lead to an accumulation and pigmentation of the skin (135).

### Vitiligo

Patients with hypothyroidism may also develop vitiligo, which is an autoimmune destruction of melanocytes resulting in areas of pigmentation and areas of hypopigmentation. It is estimated to have a prevalence of 0.5-2% of the general population. In a systematic review and meta-analysis of 77 studies between the years 1968-2018, the highest prevalence of thyroid disease in association with vitiligo was subclinical hypothyroidism (0.06% prevalence), overt hypothyroidism (0.03% prevalence), and Hashimoto’s thyroiditis (0.02% prevalence) (136). Treatment of vitiligo may initially include topical corticosteroids with or without vitamin D3 analogs. Additional treatment options include systemic steroids, topical calcineurin inhibitors, topical L-phenylalanine, topical antioxidants, and natural sunlight with oral khellin supplementation (137).

## Myxedema

While xerosis cutis may be the most common skin finding in hypothyroidism, the most classical finding is myxedema, also known as thyroid dermopathy. While more often seen in Graves’ disease, 10% of cases of myxedema result from patients with hypothyroidism and more frequently when associated with autoimmune disease. In hypothyroidism, myxedema presents as swollen, doughy, and and waxy to touch. It is more likely to develop if hypothyroidism has persisted for a long time (124). Myxedema appears as swollen, waxy, or doughy skin. Patients with myxedema have non-pitting edema that is located periorbital, pretibial, and the hands and feet (124, 138). Facial appearance may change and demonstrate swollen lips, a broad nose, macroglossia, and puffy droopy eyelids (12). Other manifestations include macroglossia, broadening of the nose and thickening of the lip (139 **)**.

Myxedema due to hypothyroidism is associated with normal or inactive dermal fibroblasts due to systemic infiltration of glycosaminoglycans chondroitin sulfate and hyaluronic acid. These glycosaminoglycans proteins then accumulate in the subendothelial later of vascular capillaries and lymphatics, leading to loss of plasma protein into the interstitial space. Additionally, glycosaminoglycans have the ability to swell in size through water retention up to 1000x its original size. The pathophysiology of myxedema is not clearly understood but it is attributed to prolonged stimulation of fibroblasts by TSH, similar to the mechanism of TSH receptor antibodies in Graves’ disease causing myxedema (124).

Due to the benign and often mild symptoms, most patients do not require any specific treatment other than thyroid hormone replacement. In these scenarios, myxedema will resolve over time. Response depends on the size of the lesion, with larger lesions being more resistant to treatment (12).

Despite frequently being associated with mild symptoms, a variety of severe manifestations of myxedema are often found more in conjunction with Hashimoto’s thyroiditis. There has been documentation in the literature of ptosis due to loss of sympathetic nerve stimulation of the superior palpebral muscles (124). In another case, a 59-year-old male developed an erythematous rash associated with limb weakness that was concerning for dermatomyositis. In this case, a skin biopsy was performed that revealed lymphocytic infiltration around the small blood vessels in the dermis along with mucin deposition. A follow up EMG did not show any signs of myositis, and additionally no other classical findings of dermatomyositis were evident. Further workup revealed positive thyroperoxidase and thyroglobulin antibodies, thus confirming the diagnosis of Hashimoto’s thyroiditis. It was determined that the patient’s skin manifestations were more akin to thyroid dermopathy. The patient’s muscle weakness and erythematous rash improved with treatment of levothyroxine and normalization of his thyroid function tests (140).

## Nails

**Figure 8:**
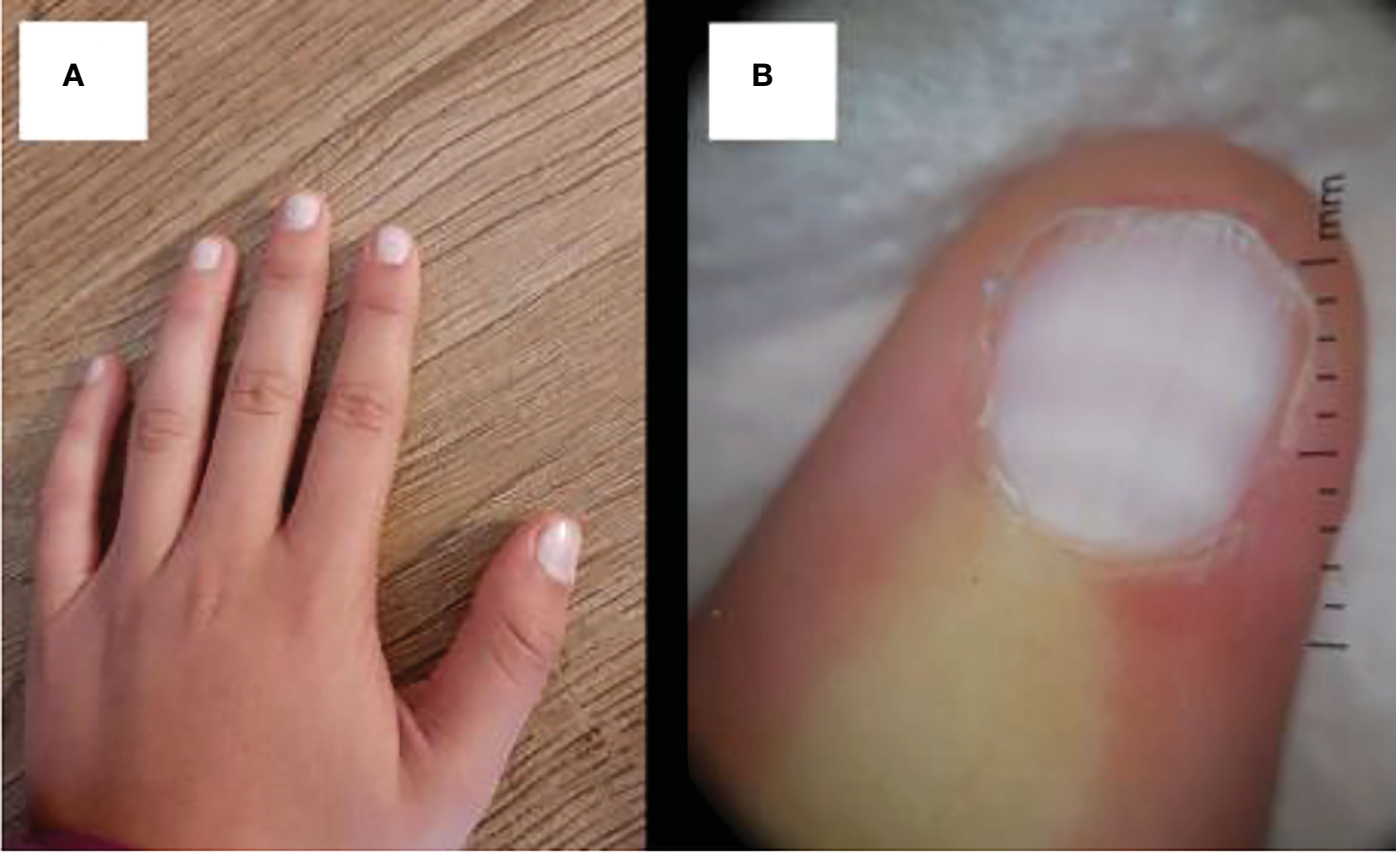
**(A)** Uniform whitish discoloration of all fingernails with disappearance of the lanula and **(B)** dermatoscopy of the nail bed, showing uniform whitening with slightly hyperchromic transversal streaks.

Multiple studies have demonstrated a variety of nail changes that occur in hypothyroidism. In a systematic review of nail changes in hypothyroidism identified that the most common manifestation was increased fragility (70%; n=50), followed by slow growth (48%; n=50), thinning of the nail (40%; n=50), onycholysis (38%; n=50), and brittleness (13.9%; n=173). Less common nail changes in hypothyroid patients were leukonychia (9.4%; n=32), striped nails (6%; n=50), and pitting (1.2%; n=173). In all cases, treatment with thyroid hormone replacement resulted in resolution of nail disease (141). An additional rare manifestation of nail disease in hypothyroidism is seen in a case report of a 12-year-old female with a past medical history of familial hyper-CK-emia, a condition where patients have familial inheritance of elevated creatinine kinase without any neurologic manifestations and with normal physical examination. This patient developed complete whitening of the nail bed of all of her fingers with disappearance of the lunula with slightly hyperchromic transversal streaks, consistent with an etiology known as Terry’s nails (**Figure 8**). The patient was found to have subclinical hypothyroidism with a TSH of 6.27 mIU/L and normal levels of FT4 and total T3. Anti-thyroglobulin and anti-thyroperoxidase antibodies were elevated consistent with Hashimoto’s thyroiditis. No other cause for her nail disease was discovered (142). There was no further documentation of treatment at that time and if it had any impact on her nail disease.

## Hair

Some of the most striking effects of hypothyroidism seen inpatients is its impact on the hair cycle. Hypothyroidism has been associated with a variety of different types of changes related to hair and can affect hair at any site in the body. The most common manifestations of hair change in hypothyroidism are alopecia and trichodystrophy, which includes dry, coarse, brittle, and slow growing hair that falls out easily. Additionally, alopecia develops due to increases in the amount of hair in the telogen phase of the hair cycle. This is known as telogen effluvium and may result in either diffuse or partial alopecia. Patients also may develop madarosis, a specific form of alopecia with loss of the lateral third of the hair of the eyebrow and loss of eyelashes. When initiating treatment of hypothyroidism, patients may initially have worsened alopecia as more hair is lost as follicles transition from telogen to anagen (143).

### Telogen effluvium

Telogen Effluvium is a type of non-scarring alopecia which occurs due to diffuse shedding of telogen club hairs. It is a condition that leads to premature entry of the hair into telogen phase. Growing human scalp follicles are sensitive to even slight fluctuations in thyroid hormones. T4 and T3 are responsible for increasing mitochondrial energy metabolism in human scalp hair follicles (144, 145). Thus, in hypothyroidism, alopecia can occur also due to prolonged telogen effluvium in addition to hair being dry and brittle. In a study by Deo et al., in a group of females with telogen effluvium, 9.63% of all subjects had hypothyroidism. Of all the subjects enrolled, 6.7% of women were newly diagnosed with hypothyroidism. In this study, it mentioned that telogen effluvium is linked to the amount of thyroid hormone in the bloodstream and not the autoimmunity in relation to the thyroid (146).

Treatment of alopecia related to hypothyroidism often resolves with initiation of thyroid hormone replacement (147). Despite commercial popularity, no clear evidence supports the use of biotin as a treatment for hair loss of any cause except in the case of a true biotin deficiency (148). Interestingly, it may still be necessary to consider other micronutrients involved in hair growth, such as zinc, as demonstrated in one case report of alopecia. In this case, a 28-year-old female presented with fatigue, xerosis, diffuse alopecia and annular scaly and erythematous plaque on the nape of the neck. She was found to have hypothyroidism with TSH 64 IU/ml (0.3-4.5) and low T3 and T4. The patient was started on levothyroxine but her dermatologic symptoms did not improve. Therefore, since the annular scaly lesion did not improve and biopsy was non-diagnostic, a plasma zinc level was evaluated, which demonstrated a plasma zinc value of 62 mcg/dl (66-144 mcg/dl) indicating a zinc deficiency. Thyroid hormone is responsible for helping to absorb zinc from our diet, and this patient had developed an acquired zinc deficiency due to hypothyroidism. Once zinc supplementation was added to her treatment plan, the patient’s alopecia started to resolve in addition to her hypothyroid symptoms (149).

## Other skin diseases associated with hypothyroidism

As mentioned earlier, the most common cause for hypothyroidism is autoimmune, secondary to Hashimoto’s thyroiditis. Therefore, it is necessary to recognize patients with autoimmune thyroid disease are at higher risk for other autoimmune disease. One such disease, is alopecia areata, a form of alopecia caused by immune mediated destruction of follicles in the anagen phase of the hair cycle. It is currently recommended that all patients with alopecia areata be screened for autoimmune thyroid disease. One additional study done of 78 adult patients with newly diagnosed alopecia areata showed abnormal thyroid function tests or anti-thyroid antibodies in 24% of patients. In that study, 15% of patients were diagnosed with subclinical hypothyroidism, 1% with overt hypothyroidism, 1% with overt hyperthyroidism, and 5% with euthyroid Hashimoto’s thyroiditis (150).

Other common conditions that may be associated with hypothyroidism, and more commonly with autoimmune thyroid disease, include the dermatologic manifestations in discoid lupus, systemic lupus erythematosus, bullous disorders, systemic sclerosis, and CREST syndrome (124).

## Thyroid cancer and skin disease

Thyroid cancer is the most common endocrine malignancy in the United States. It is three times as prevalent in females compared to males. The median age of onset of thyroid cancer is 51 years of age, which is young for many malignancies (151). Thyroid cancer that derives from follicular cells can be classified as either differentiated or undifferentiated. Differentiated thyroid cancers include papillary and follicular thyroid cancer and are much more common than undifferentiated thyroid cancer. Undifferentiated thyroid cancer includes anaplastic thyroid cancer. Thyroid cancer that derives from parafollicular cells includes medullary thyroid cancer (152).

In general, the cutaneous manifestation of non-dermatologic cancers is rare, estimated to be present in 0.7-0.9% of all cancer patients (153). This was demonstrated in a study of 4020 patients with varying types of metastatic cancer, where approximately 9% of subjects had developed some cutaneous manifestation of their malignancy (154). Regarding papillary thyroid cancer, the most common site of metastasis is to locoregional lymph nodes, and in some cases with hematogenous spread to lung, liver, bone or brain. It is estimated that cutaneous metastasis occurs in 1 per 1000 cases. Of these, 41% are from papillary thyroid cancer, 28% from follicular thyroid cancer, 15% from medullary thyroid cancer, and 15% from anaplastic thyroid cancer (155). It should be noted that while the majority of cases with cutaneous metastases came from papillary thyroid cancer, other types of thyroid cancer, specifically follicular thyroid cancer, had much higher prevalence of cutaneous spread in comparison to their much lower prevalence in comparison to other types of thyroid cancer (156). When present they often manifest as a firm subcutaneous nodule, either flesh colored or violaceous, sometimes ulcerated, and occasionally highly vascularized. The most common location for cutaneous spread is on the head and neck. The clinical significance of this is that cutaneous manifestations may be the first clue of extrathyroidal extension of a primary thyroid cancer and in rare cases may be the initial presentation of a primary thyroid cancer (124).

Diagnosis of thyroid cancer is most often made after a patient may complain of swelling or growth in the neck or if a nodule, or nodules, are found either on physical exam or other unrelated head or neck imaging modalities. In order to obtain a diagnosis, patients undergo fine needle aspiration (FNA). This technique has good specificity for thyroid cancer when performed in the appropriate clinical context. FNA is a technique used for evaluation of other types of cancer and, while generally tolerated well, seeding the needle track with malignancy presents minimal risk. A literature review of 4912 patients who underwent FNA for evaluation of papillary thyroid cancer between 1990 and 2002 found that there were a total of 7 cases (0.14%) of documented needle tract seeding of malignancy from FNA (157).

**Figure 9:**
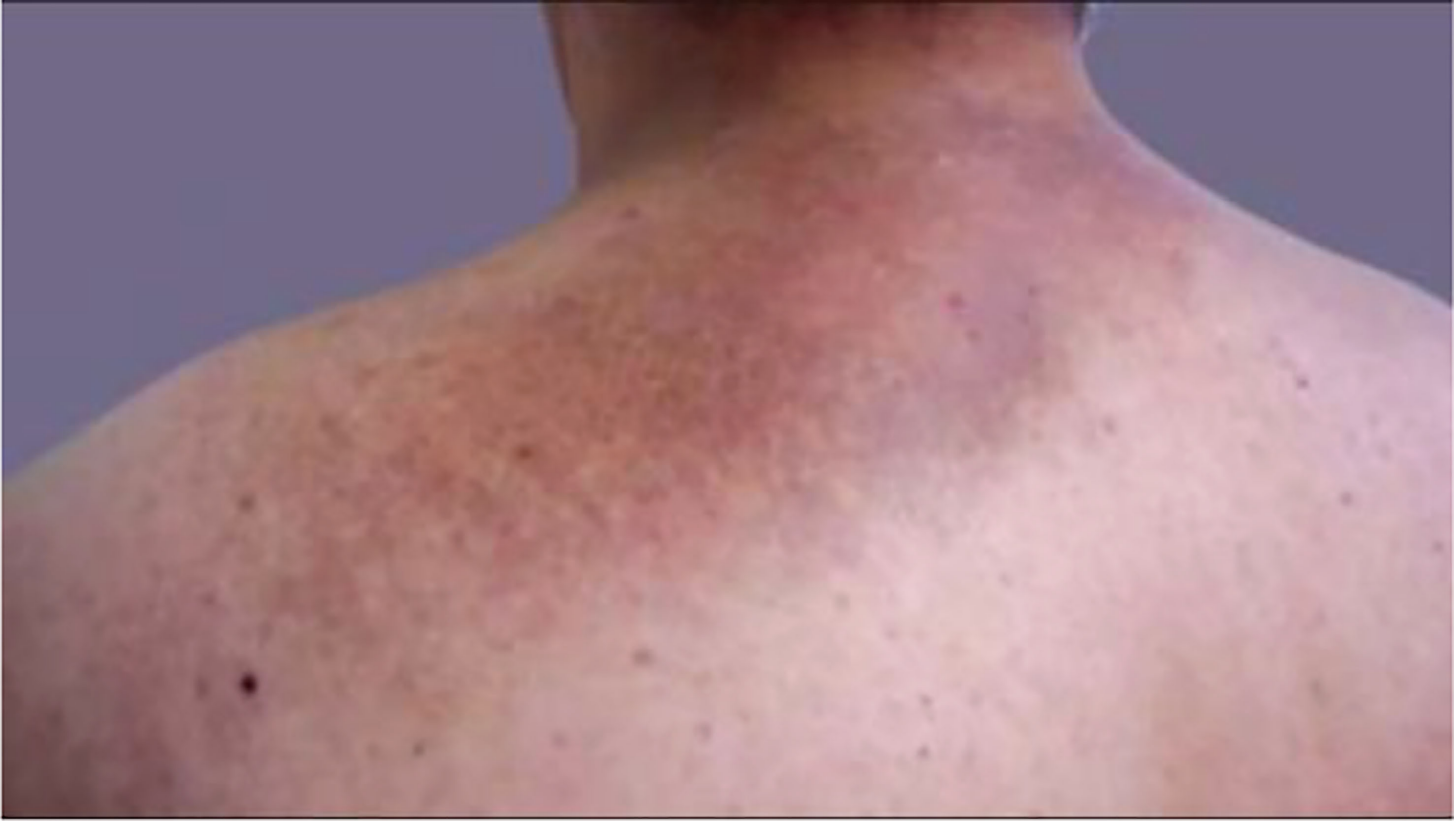
Cutaneous lichen amyloidosis presents clinically as macules, spots or pruritic hyperpigmented plaques with undulating surface (rippled patches), of a brownish or blackish color, with ill-defined borders, usually located in the interscapular region.

**Figure 10:**
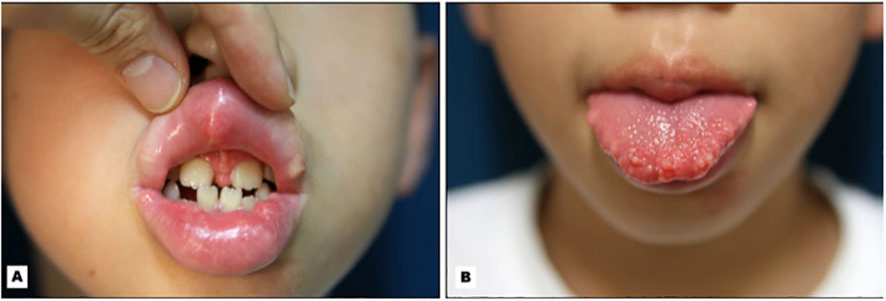
Mucosal neuromas present on the **(A)** lips and **(B)** tongue in a child with MEN2b.

Like many types of cancer, a variety of thyroid cancers are associated with a specific genetic syndrome, of which those with cutaneous manifestations of that specific disease may help in earlier screening, diagnosis, and management of thyroid malignancy.One of the most common genetic syndromes associated with thyroid cancer are the multiple endocrine neoplasia (MEN) type 2A and 2B. This is an autosomal dominant syndrome associated with medullary thyroid cancer and arises due to a mutation in the RET proto-oncogene. Mutations in different areas of the RET proto-oncogene leads to different types of mutations that may manifest as either MEN type 2A or 2B. In MEN2A, patients are at risk for medullary thyroid cancer, pheochromocytoma, and primary parathyroid hyperplasia. Cutaneous manifestations of MEN2A include cutaneous lichen amyloidosis found in the interscapular area that appears as pruritic, scaly, lichenoid papules with hyperpigmentation (**Figure 9**) (158). In MEN2B, patients are also at risk for medullary thyroid cancer and pheochromocytoma, but do not develop parathyroid hyperplasia. Instead, patients develop mucosal neuromas (**Figure 10**) (159) on the conjunctiva, lips, and tongue, as well as intestinal ganglioneuromas. Patients may also develop joint laxity and appear with a marfanoid habitus (124).

Another genetic condition with both cutaneous manifestations and thyroid cancer is familial adenomatous polyposis (FAP). This is an autosomal dominant disease due to a germline mutation in the adenomatous polyposis (APC) gene. FAP has an incidence rate of 1 in 8300 births and has been shown to affect both sexes equally. This condition manifests in hundreds and sometimes thousands of adenomatous polyps in the rectum and colon, generally in a person’s second decade of life, and leads to colorectal cancer. In addition to the colorectal manifestations, there are a variety of extracolonic manifestations. In Gardner syndrome, a variant of FAP, patients may develop a variety of dermatologic manifestations include epidermoid cysts and fibromas of the scalp, shoulder, arms, and back (160). In addition to the extracolonic manifestations seen in FAP, patients have a lifetime risk of developing thyroid cancer estimated to be 1-2%. The most common thyroid cancer associated with FAP is papillary thyroid cancer. Treatment for patients who have FAP and develop thyroid cancer is the same as the general population, and patients generally have favorable outcomes (161).

Carney complex, also referred to by either NAME (e.g., nevi, atrial myxoma, myxoid neurofibromas, ephelides) or LAMB (lentigines, atrial myxoma, mucocutaneous myxoma, blue nevi) syndrome depending on the patient’s presentations, is an autosomal dominant disease associated with a mutation in the PRKAR1A gene and is associated with multiple endocrine malignancies including thyroid cancer, Sertoli cell tumors, primary pigmented nodular adrenocortical disease, and acromegaly secondary to growth hormone secreting pituitary adenomas. About 750 cases of this disease have been reported worldwide. In patients with Carney complex it is estimated that 60% of patients have cystic or multinodular thyroid disease on ultrasound, with follicular adenomas being the most common biopsy findings (162). This was illustrated in a study of 350 patients of whom 25% were noted to have adenomatous changes to the thyroid of which 2.5% were found to develop thyroid cancer (163). Further studies have demonstrated that the thyroid cancer was associated with Carney complex. In a study of 26 patients with Carney complex and a thyroid disorder, 16 patients were found to have adenomas and 10 patients were found to have carcinoma of which three were papillary thyroid cancer and seven identified as follicular thyroid cancer. Additionally, it was noted that some of these follicular thyroid cancers were large, greater than 3 cm and had already metastasized (4 out of 7 cases), and multifocal and bilateral (2 out of 7 cases). Follicular thyroid cancer was fatal in 3 out of 7 cases and was demonstrated to be resistant to treatment with radioactive I (127) or to a tyrosine kinase inhibitor. It should be noted that in cases of Carney complex, patients are more likely to develop follicular, rather than papillary thyroid cancer (162). Given the high incidence of thyroid nodules and thyroid cancer in these patients, performing routine thyroid sonography to monitor for risk of development of thyroid cancer may be reasonable, although no current guidelines outline a timing for initiation of screening or interval frequency of which this may be beneficial.

**Figure 11:**
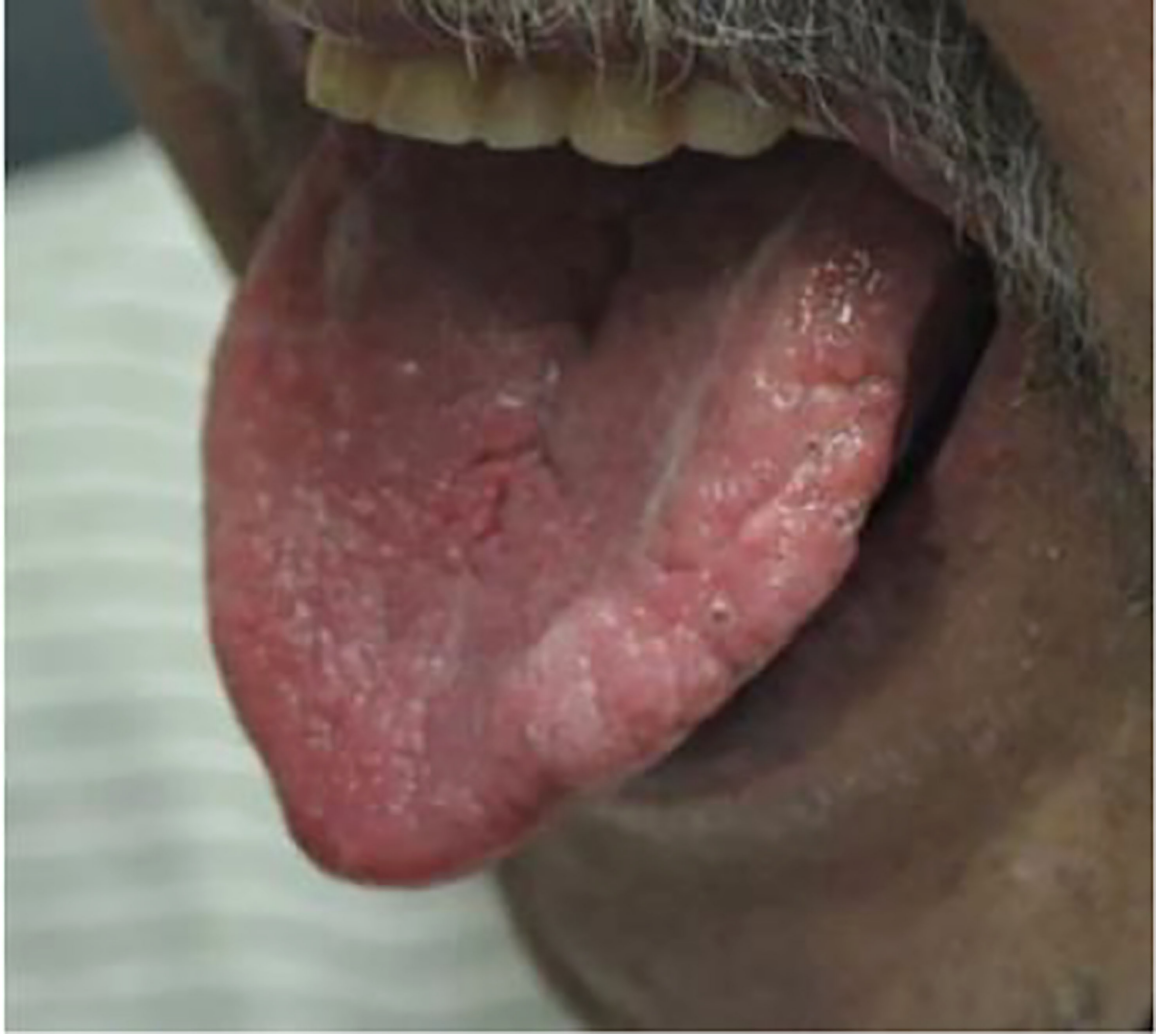
Oral papillomas.

**Figure 12:**
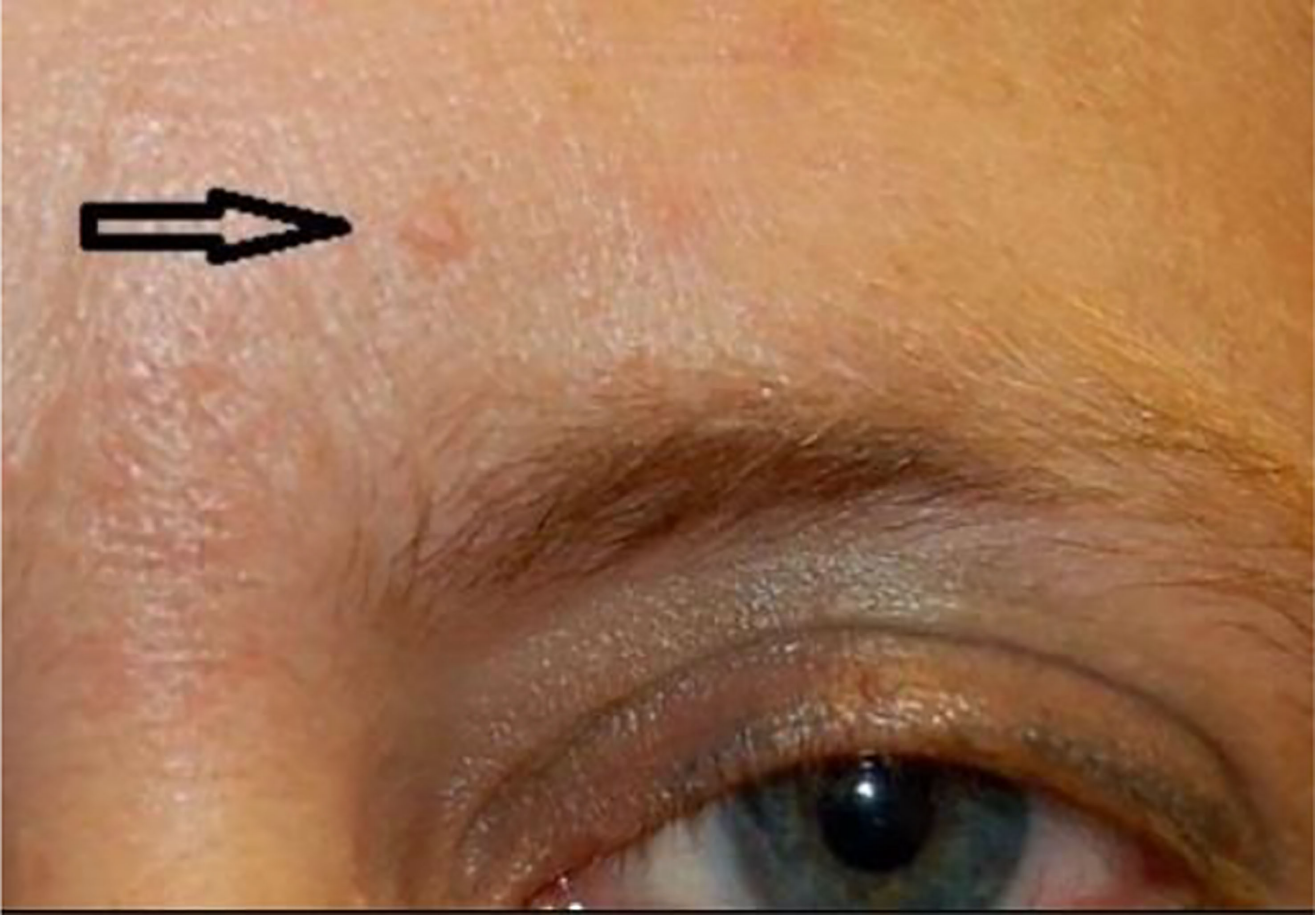
Trichelemmoma.

**Figure 13:**
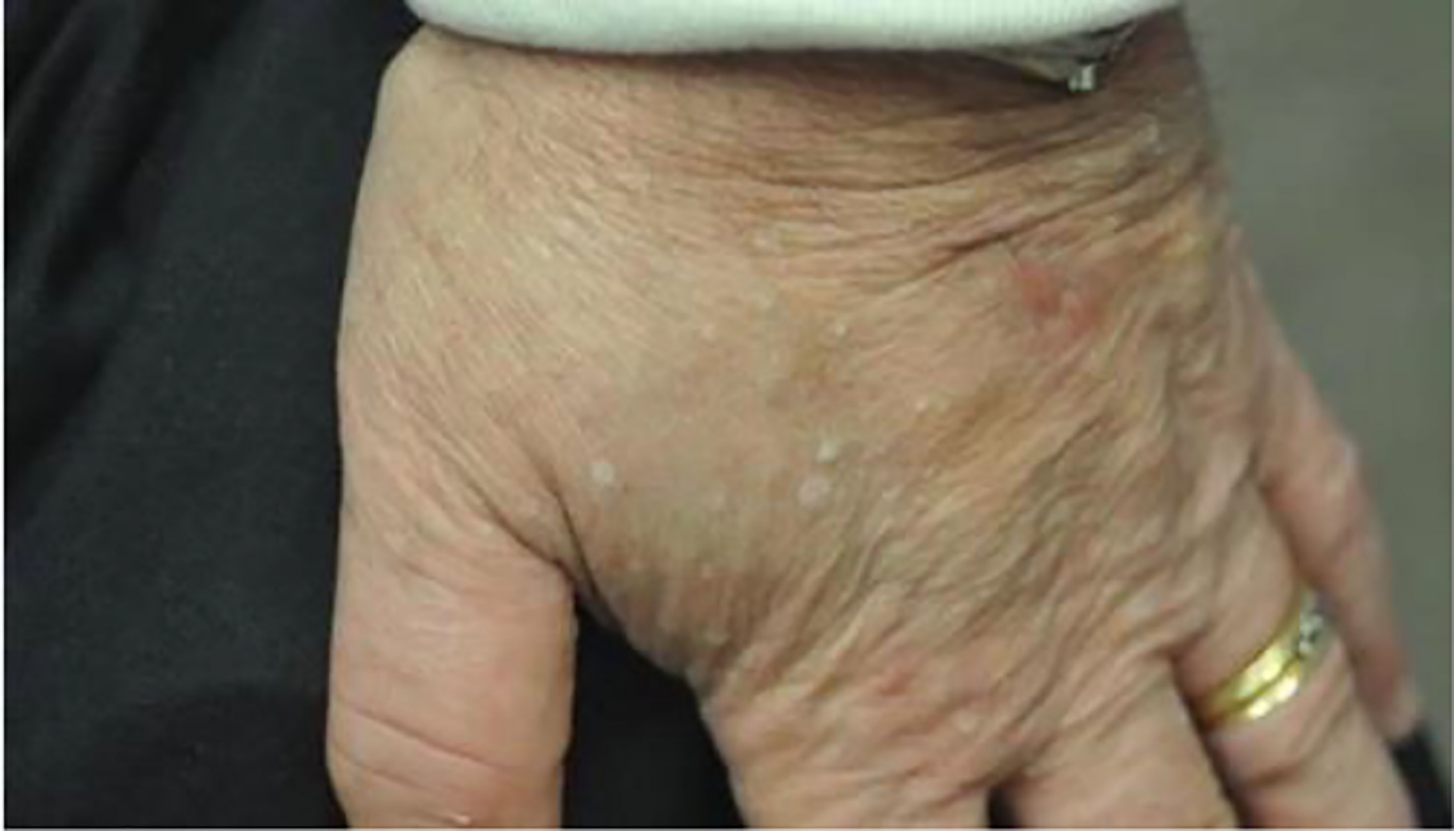
Acral keratosis.

Lastly, Cowden syndrome is an autosomal dominant hamartomatous disease that develops due to mutation in the PTEN gene (164). The overall incidence of Cowden syndrome is currently estimated to be 1 in 200,000 to 250,000 (165). PTEN is a negative regulator of a phosphoinositide-3-kinase (PI3K)-AKT pathway and the increased risk for oncogenesis when this loss-of-function mutation occurs. Cowden syndrome therefore presents with multiple hamartomas and malignancies. The initial presentation is often due to cutaneous manifestations that include: (1) facial and oral papules that are smooth (**Figure 11**) (165), white, lesions 1-4 mm in diameter, (2) trichilemmomas (**Figure 12**) (164), hamartomatous tumors of the outer root sheath of the hair follicle, and (3) acral keratosis (**Figure 13**) (166), which are 1-4 mm keratotic verrucous papules on the dorsal hands, wrists, or feet. Patients with Cowden syndrome are also at risk for developing multinodular goiter, Hashimoto’s thyroiditis, and are estimated to have a 70-fold increased risk of thyroid malignancy compared to the general population and an incidence rate of 3 to 38% depending on the study (167). Thyroid cancer is the second most common malignancy in patients with Cowden syndrome (168). Due to the increased risk of thyroid cancer, the National Comprehensive Cancer Network recommends initial screening for thyroid cancer with thyroid ultrasound starting at seven years of age with repeat screening imaging annually (167).

## Conclusion

The knowledge regarding skin manifestations associated with thyroid disease and their etiology has increased considerably in the last decade. Various cutaneous manifestations are seen with overt thyroid hormone imbalance. Clinicians that are equipped with the knowledge of various skin pathologies will be able to make a more expeditious diagnosis of thyroid disease that can easily be performed through routine thyroid function studies. Thyroid hormone replacement generally will treat the underlying skin disease that is seen in hypothyroidism. Treatment in hyperthyroidism either with thionamide therapy, surgery, or radioactive iodine usually will assist with generating a euthyroid state as well as clearing the skin disease associated with it. Other skin diseases that are seen in conjunction with other autoimmune diseases are more challenging in terms of treatment options. However, given the complexity of the molecular mechanisms with thyroid hormone disorders and cutaneous manifestations, much remains to be unraveled.

## Author contributions

BC is the lead author on the manuscript, performing editing and formatting of the entire document as well as writing up the sections on hypothyroidism and thyroid cancer. AC wrote the sections on hyperthyroidism and chronic and acute urticaria, as well as compiled the figures. SJ is the senior author on the paper, and helped supervise and guide both BC and AC, as well as help with editing and writing the abstract and introduction. All authors contributed to the article and approved the submitted version.
